# Evaluating the effect of interactive two-way texting on 6-month antiretroviral therapy outcomes: Findings from a randomized controlled trial in Lilongwe, Malawi

**DOI:** 10.1371/journal.pgph.0004598

**Published:** 2025-09-10

**Authors:** Christine Kiruthu-Kamamia, Robin E. Klabbers, Hannock Tweya, Jacqueline Huwa, Agness Thawani, Pachawo Bisani, Joseph Chintedza, Geldert Chiwaya, Aubrey G. Kudzala, Dumisani Ndhlovu, Johnnie Seyani, Wim Groot, Milena Pavlova, Caryl Feldacker

**Affiliations:** 1 United Nations University—Maastricht Economic and Social Research Institute on Innovation and Technology, Maastricht, Netherlands; 2 Lighthouse Trust, Lilongwe, Malawi; 3 International Training and Education Center for Health, Seattle, Washington, United States of America; 4 Department of Emergency Medicine, University of Washington, Seattle, Washington, United States of America; 5 Department of Global Health, University of Washington, Seattle, Washington, United States of America; Boston University, UNITED STATES OF AMERICA

## Abstract

Retention on antiretroviral therapy (ART) is critical for achieving viral load suppression (VLS) among people living with HIV (PLHIV). Retention remains challenging in high-prevalence settings like Malawi. Short messaging service (SMS) interventions, particularly hybrid two-way texting (2wT), show promise in improving ART retention. We conducted a randomized control trial (RCT) at Lighthouse Trust in Lilongwe, Malawi, to evaluate the effectiveness of a hybrid 2wT system to improve compliance to ART refill appointments, early retention, and VLS among new ART initiates. After receiving routine ART initiation counseling, 442 clients with mobile phones on ART ≤ six months were randomized to 2wT or standard of care (SoC). The 2wT group received weekly motivational messages, appointment reminders, and access to an open-ended SMS communication channel with retention officers. The SoC group received peer support at clinic visits and reminder calls, pre-visit and for missed appointments. All participants were traced if they missed an appointment by >14 days. Study outcomes ascertained six months post ART initiation included: ART appointment compliance (attending within 2 days), retention in care (alive on ART), and VLS (< 200 copies). Data from electronic medical records were analyzed using Chi-square tests and multivariable logistic regression. Data on 2wT participation were extracted from the 2wT database and summarized using Microsoft Excel. At six months, the 2wT group demonstrated higher compliance to ART refill visits compared to SoC (59.6% vs. 46.8%, p = 0.008). Among 2wT participants, 86% responded to ≥ 1 message, suggesting strong engagement with 2wT. The platform sent 1,645 reminders: all participants received visit reminders with the majority receiving multiple pre-appointment and follow-up messages. However, 2wT did not significantly improve retention or VLS at 6 months. Over 91% of both 2wT and SoC were retained in care (p = 0.969), and higher VLS among 2wT (97.5%) vs. SoC (93.2%) was not significant (p = 0.07). Implementing a larger 2wT study over 12–24 months and among more clients could provide evidence of 2wT impact on retention and VLS. Overall, the enhanced ART visit compliance and high care engagement among 2wT participants suggests that the low-tech, highly-accepted, 2wT approach could provide a scalable, appropriate complement to existing retention interventions in low-resource settings.

## Background

While significant global progress has been made in initiating people living with HIV (PLHIV) on antiretroviral therapy (ART), retaining PLHIV on ART remains a challenge, especially during the first 12 months [1–3]. Retention is pivotal in achieving viral load suppression (VLS) [4,5]. Malawi, with an HIV prevalence rate of 6.7% among adults [6], is progressing: 88% of PLHIV were aware of their status, 98% of those PLHIV who were aware of their status were on ART, and 97% of PLHIV on ART achieved VLS [7]. Still, challenges persist in retaining individuals in ART care over time [8,9], particularly in the first year of treatment. Studies in Malawi show that a substantial proportion of new ART initiates are lost to follow-up (LTFU) within the first year, driven by structural barriers (e.g., transport costs, long wait times) [1], psychosocial factors (e.g., stigma, lack of disclosure) [10,11], and health system limitations (e.g., limited provider capacity) [3,9,12]. These early gaps in retention jeopardize long-term VLS gains and highlight the need for feasible, low-cost strategies to support clients during the initial treatment period.

The use of short messaging services (SMS) shows promise to enhance ART medication adherence, provide ART refill appointment reminders, and facilitate communication with healthcare workers [13–16]. Evidence suggests that interactive two-way messaging, which allows for messages with response options between clients and healthcare workers, is more effective than one-way informational messaging in improving ART-related care outcomes [13,17,18].

In 2021, the Lighthouse Trust Martin Preuss Center ART clinic in Malawi, in collaboration with the International Training and Education Center for Health (I-TECH) at the University of Washington and technology partner Medic, conducted a quasi-experimental study to design, develop, test, and assess an interactive two-way texting (2wT) system to improve early retention among new ART initiates [19–21]. Lighthouse Trust implements an early retention Differentiated Service Delivery (DSD) model for new ART clients during their first 12 months on ART, providing targeted support during this critical period. In the quasi-experimental study, we prospectively followed a cohort of newly initiated ART clients who received the 2wT intervention and compared their outcomes to a matched, historical comparison group of clients who had initiated ART one year earlier under standard of care (SoC). The study found that 2wT demonstrated high usability and acceptability for both clients and healthcare workers [19,22], improved ART retention at 12 months by 15% [23], and appeared cost-effective when considered at scale [21].

To strengthen the quasi-experimental findings, we conducted a small, pragmatic, randomized controlled trial (RCT) among only new ART clients with mobile phones at Lighthouse Trust ART clinics in Lilongwe, Malawi. The RCT aimed to evaluate the 2wT effect on three outcomes at 6 months post-ART initiation: 1) compliance to ART refill visits, 2) retention on ART, and 3) VLS. We hypothesized that 2wT clients would show higher ART refill visit compliance, retention, and VLS at 6 months post ART initiation compared to SoC peers.

## Methods

### Setting

Lighthouse Trust (LT), a WHO-recognized Center of Excellence for HIV care, has partnered with the Malawi Ministry of Health (MoH) since 2001 to operate public ART clinics [24–26]. LT operates two clinics in Lilongwe: Lighthouse Clinic at Kamuzu Central Hospital and Martin Preuss Center at Bwaila Hospital. These clinics provide free HIV care to over 38,000 clients and start over 500 clients on ART quarterly. Newly diagnosed clients start ART the same day and are registered in the Electronic Medical Records System (EMRS). Clients have monthly ART refill visits for six months. Stable clients then transition to 3-monthly visits, while less stable clients continue monthly visits until assessed stable and, therefore, eligible for multi-month dispensing. Viral load (VL) testing is conducted six months post-ART initiation and then annually thereafter. The EMRS records visits, medications, side effects, adherence to ART, visit attendance, VL results, and ART outcomes.

### Study design, recruitment, and enrollment

Participants in this two-arm, prospective RCT were recruited between December 16, 2022 and June 28, 2023. Eligibility criteria included recent ART initiation (within 2 months), aged 18 years or older, mobile phone ownership, basic literacy, and verification of enrolment message receipt on the mobile phone. Consenting participants were randomized 1:1 to either the 2wT (intervention) or SoC (control) using a block design in groups of 10. Hoping to replicate the improved retention findings from the quasi-experimental study [23], but acknowledging less follow-up time, we required 394 participants (197 per arm) to achieve 80% power to detect a meaningful 10% point improvement in retention at 6 months (the primary outcome from the quasi-experimental design), assuming 80% retention in the control group based on routine program data. The sample size was determined using a log-rank test–based power calculation. Accounting for potential 10% attrition, 410 (205 per arm) were needed. We aimed to enroll 500 participants to allow for buffer and withdrawal of those documented lack of SMS confirmation receipt. A total of 467 participants were enrolled and followed for 6 months post-ART initiation. 2wT participants chose Chichewa or English messages; all SMS communication was free of charge. The study was registered with the Clinical Trial Registry on 9/6/2022 (NCT05531448). The Consolidated Standards of Reporting Trials (CONSORT)Outcomes 2022 checklist [27] was used in conducting the study (S1 Table).

### The study interventions

#### Standard of care (SoC).

SoC clients received early retention support through expert client (EC) buddies and late retention support through the Back to Care (B2C) tracing program. For the first year on ART, SoC clients were supported by an EC buddy who is a PLHIV. EC buddies offered in-clinic ART adherence counseling, called clients with phones to provide appointment reminders, and followed-up with calls within 14 days of a client missing an appointment [21,23]. Fourteen days after a missed visit, clients were referred to the B2C tracing program, where the B2C team called or visited clients to return them to care [28].

#### Two-way texting (2wT).

The 2wT system, detailed previously [19,22,23,29], was designed as a complement to EC buddy support which is labor and time-intensive. 2wT clients received automated, weekly motivational messages, individualized SMS appointment reminders three days and one day before a scheduled appointment, and (if needed) SMS reminders 2, 5, and 11 days after a missed appointment. Additionally, 2wT accommodated open-ended SMS communication between clients and retention officers to reschedule visits, report transfers, or discuss non-clinical issues related to their ART care. Currently, 2wT support continues indefinitely if 2wT clients do not stop messages. Like SoC clients, 2wT clients are also referred to B2C 14 days after a missed appointment. 2wT clients do not receive EC buddy support. Those who opted out of 2wT after receiving 2wT services were classified as withdrawn.

### Data collection

Data on MoH ART outcomes, compliance to ART refill visits, and VLS for both the intervention and control groups were extracted from the EMRS for the first six months post-ART initiation. MoH ART outcomes include: alive in care (individuals retained in ART care as of the date of record review), stopped ART treatment (notified the clinic of their decision to stop treatment), transferred out (documented relocation), died (death due to any cause), LTFU (did not return to the clinic within 60 days of a scheduled visit) [30] and withdrawn (withdrawn from the study after enrolment). Clients who were lost but returned to care before the 6-month endpoint were considered alive in care at that time. As implemented in the quasi-experimental study, RCT participant engagement data, including message delivery, responses, and interaction patterns, were downloaded from the 2wT database and aggregator service [31].

### Study outcomes

Primary study outcomes measured at 6 months post-ART initiation included: 1) compliance to ART refill visits defined as attended within 2 days of the scheduled appointment; (2) retention defined as the proportion of clients retained in care (alive in care) at 6 months post ART initiation regardless of prior missed appointments; and 3) VLS defined as, suppressed = HIV RNA < 200 copies. Secondary outcomes on 2wT platform engagement included message delivery success, responses to scheduled reminders, and client-initiated messages.

### Statistical analysis

2wT participants were classified as exposed to 2wT and included in the analysis if they received at least one reminder SMS during the 6-month follow-up. All analyses were conducted on an intention-to-treat basis, where participants were analyzed in their originally assigned groups regardless of their level of engagement or withdrawal. Descriptive statistics compared participant characteristics and baseline WHO stage. Risk differences were used to compare compliance to ART refill visit, MoH ART outcomes, and VLS at 6 months post-ART initiation between the study arms. Since VL data was not available for some participants, we assessed whether having missing VL data was associated with baseline characteristics using logistic regression. A multivariable logistic regression analysis was used to estimate the odds of achieving VLS, adjusting for age, sex, intervention arm, ART refill visit compliance, and WHO stage at ART initiation. The effect of the intervention on retention was assessed by comparing six month proportions using a chi-square test. Since VL data was not available for some participants, we also assessed whether having missing VL data was associated with baseline characteristics using logistic regression. Time-to-event analysis was used to estimate retention probabilities over time and the effect of the intervention. LTFU and stopped treatment were considered failure events. Participants who were still in care were censored 6 months after ART initiation, accounting for accrued time on ART for those joining after ART initiation. Kaplan-Meier plots were used to display retention probabilities, comparing 2wT and control groups using log-rank tests. Participants who withdrew from 2wT were excluded. We also conducted post-hoc mediation analysis using 5,000 bootstrap replications to assess whether ART refill visit compliance mediated the pathway between 2wT intervention and VLS.

### Ethical consideration

The study protocol (S1 Protocol) received approval from both the Malawi National Health Sciences Research Committee (#22/10/3036) and the University of Washington, Seattle, USA, ethics review board (STUDY00010106). All participants provided written informed consent in either Chichewa or English before enrolling in the study.

## Results

Included and excluded clients and the reasons for their exclusion are represented in the consort flow chart (S1 Fig). Clients were evenly distributed between clinics (Table 1). About half of all participants were female; most had WHO clinical stages 1 or 2 conditions at ART initiation; and the median number of scheduled visits during the study was 3 (Interquartile range [IQR] 2.0-4.0).

**Table 1:** Participant characteristics of clients enrolled in SoC and 2wT.

	SoC N = 228		2wT N = 214		P-value
	N	%	N	%	
**Study site**					
LH	63	27.6%	58	27.1%	0.901
MPC	165	72.4%	156	72.9%	
**Sex**					
Male	118	51.8%	98	45.8%	0.21
Female	110	48.2%	116	54.2%	
**Age (Median [IQR])**	35	[28-42.5]	36	[29–42]	
**Age group**					
18–19	6	2.6%	3	1.4%	0.59
20–24	22	9.6%	17	7.9%	
25–29	39	17.1%	42	19.6%	
30–34	42	18.4%	34	15.9%	
35–39	46	20.2%	37	17.3%	
40–44	31	13.6%	42	19.6%	
45–49	21	9.2%	23	10.7%	
50+	21	9.2%	16	7.5%	
**WHO HIV stage**					
1 or 2	167	74.2%	140	66.4%	0.085
3	43	19.1%	45	21.3%	
4	15	6.7%	26	12.3%	

2wT = two-way texting; SoC = Standard of care; LH = Lighthouse Trust; MPC = Martin Preuss Center; IQR = interquartile range; WHO = World Health Organization

### ART refill visit compliance

Overall ART refill visit compliance across all 6 months was significantly higher in the 2wT group, with 59.6% of participants adhering to all scheduled ART refill visits compared to 46.8% in the SoC group (p = 0.008)(Table 2). The majority of the scheduled visits occurred within 2 days in both groups. At the individual visit level, the 2wT group also had higher visit compliance (81.1%) as compared to SoC peers (74.4%; pairwise chi-square test, p = 0.02)

**Table 2:** ART refill visit compliance by study arm at 6 months.

	SoC		2WT		P-value
	N	%	N	%	
**Overall ART refill visit compliance (patient-level)**					^*^0.008
Yes	103	46.8%	127	59.6%	
No	117	53.2%	86	40.4%	
**Number of visits**	640		618		**–**
Median visits scheduled	3 [2.0-4.0]		3[2.0-4.0]		
**ART refill visit (visit-level)**					^*^0.02
^**^Returned on time (within 0–2 days of an appointment)	477	74.5%	501	81.1%	
Returned late (3 + days after an appointment)	154	24.1%	111	18.0%	
Never Returned	9	1.4%	6	1.0%	

* chi-square association;

** pairwise chi-square test =0.005

### ART retention at 6 months

Six months after ART initiation, retention was similar between groups: 91.1% of 2wT participants were retained (alive and on ART), compared to 91.2% in SoC (p = 0.969) (Table 3). The Kaplan-Meier curves revealed no difference in retention on ART over time between 2wT and SOC (p = 0.4) (S2 Fig). Compared to SoC, 2wT trended towards a lower proportion of participants LTFU (2.8% vs. 3.9%) and a higher proportion of clients who transferred out (4.2% vs. 3.5%) and died (1.9% vs. 0.9%) at six months, but these differences were not statistically significant. No participants were withdrawn from the study.

**Table 3:** Association of intervention arm with ART outcomes and viral load suppression at 6-months.

	SoC N = 228		2wT N = 214		Risk difference (95% CI)
	N	%	N	%	
**ART outcomes**					
^*^Alive and on ART	208	91.2%	195	91.1%	‒0.1% (‒0.5–0.5)
LTFU	9	3.9%	6	2.8%	‒1.1% (‒4.3–2.2)
Stopped treatment	1	0.4%	0	0.0%	‒0.4% (‒1.3–0.4)
Transferred	8	3.5%	9	4.2%	0.7% (‒2.8–4.1)
Died	2	0.9%	4	1.9%	1.0% (‒1.2–3.1)
^**^ **Viral load**	N = 161		N = 158		
Suppressed (<200 copies)	150	93.2%	154	97.5%	
Unsuppressed	11	6.8%	4	2.5%	–4.3% (–0.3–8.9)

ART=antiretroviral treatment; LTFU=lost to follow up

* retention (alive on ART): p= 0.969

** data missing for 123 clients (67 and 56 clients at SoC and 2wT, respectively)

### VLS at 6 months

Of the 442 participants, 319 (72%) had VL measured at 6 months. Among those 319, VLS trended higher among 2wT (97.5%) compared to SoC (93.2%); however, VLS did not differ significantly between groups (p = 0.07). Of the 123 (28%) who did not have VL measured at six months (S2 Table), 85 (68%) were alive on ART at 6 months, while the remaining 39 had disengaged from care before six months: 15 (12%) were LTFU, 1 (1%) stopped treatment, 17 (14%) transferred out, and 6 (5%) died. There were no significant differences between participants with and without VL in terms of age, age or WHO stage at initiation. In the adjusted logistic regression analysis controlling for age, sex, intervention arm, ART refill visit compliance, and WHO stage (Table 4), there we no significant associations between 2wT and VLS at 6 months. In a post hoc analysis to explore possible mediation of the effect of 2wT on VLS through visit compliance, 2wT significantly increased visit compliance (p = 0.027); however, visit compliance showed a non-significant trend toward improving VLS (p = 0.088). Thus, in this analysis, visit compliance was not a mediator in the relationship between 2wT and VLS.

**Table 4:** Logistic regression analysis of achieving viral load suppression at 6 months.

Variable	Adjusted Odds Ratio*	p-value	95% Confidence interval
Intervention Arm	2.74	0.10	0.83–9.03
Overall Compliance	2.60	0.10	0.84–8.01
Female	0.34	0.09	0.10–1.17
Age 35+	1.08	0.89	0.36–3.27
WHO Stage 3	1.42	0.66	0.29–6.90
WHO Stage 4	0.86	0.89	0.10–7.53

WHO-World Health Organization

*adjusted for age, sex, intervention arm, ART refill visit compliance, and WHO stage

### 2wT Platform Engagement

Among the 214 participants enrolled in the 2wT arm, the vast majority (185 (86%)) showed high engagement with the 2wT system by either responding to or initiating messages. Most (62%) 2wT users sent more than three messages (Table 5). The platform sent 1,645 reminders, with all participants receiving a three-day reminder, 98% received a one-day reminder. Of those who missed a visit, 76%, 42%, and 13% received follow-up reminders two, five, and eleven days after their missed visits, respectively (S3 Table).

**Table 5:** Use of two-way communication by 2wT participants (n = 214).

Total number of prompted and unprompted messages sent by participants to the 2wT platform during the study period	Participants (N = 214) N(%)
0 messages	29 (14%)
1 message	16 (7%)
2 messages	22 (10%)
3 messages	15 (7%)
>3 messages	132 (62%)

## Discussion

Our pragmatic RCT investigated the effect of 2wT on compliance to ART refill appointments, early retention, and VLS among new ART initiates with phones during the first six months of ART at LT clinics in Lilongwe, Malawi. 2wT participation was associated with improved ART refill visit compliance. 2wT platform engagement was high. Likely, the personalized SMS reminders, motivational messages, and open communication channel provided by 2wT enhanced ART refill visit compliance among new ART initiates with phones who elected to participate. These findings align with previous research on SMS interventions with a positive impact on ART refill visit compliance [13,15,17,18,32]. However, ART retention and VLS at 6 months did not differ significantly between intervention groups, in contrast to previous studies [13,33]. This may be due to weaknesses in the intervention, itself, or the short RCT follow-up time and small sample size. While this RCT did not demonstrate statistically significant improvements in 6-month retention or VLS, the improvement in ART refill visit compliance among 2wT participants and high levels of participation suggests that 2wT may be a beneficial, complementary intervention to support clients’ engagement in early ART care. We discuss several implications of these findings.

First, although 2wT did not improve retention or VLS at 6 months in this study, 2wT did increase on-time visit attendance. This reduces retention officer efforts to pull files and call clients immediately after a missed visit. In addition to reductions in workload, 2wT is likely worth scaling as long-term ART visit compliance is associated with VLS [34,35]. 2wT modeling also shows that it would require fewer financial and human resources to keep clients attending their visits on time, especially at scale [21]. Leveraging those findings of timely ART refill visits and cost-benefits of 2wT at scale, the potential to reduce demand on scarce human tracing resources becomes increasingly attractive. Every missed visit triggers a labour-intensive, early client tracing effort by early retention teams, including file tracking and phone calls. If more clients attend their visits on time, that will free retention officer time to trace those without phones or those who miss visits by >14 days, better allocating scarce resources. With similar retention rates and lower documented implementation costs, 2wT scale-up should be recommended for interested individuals with basic literacy and access to a phone, freeing human and financial resources to sustain both 2wT and B2C tracing implementation [23].

Second, high engagement with the 2wT platform suggests that the interaction makes clients feel cared for and supported, as shown previously [20]. The combined motivational messages and the individualized visit reminders likely improve participant self-efficacy and motivation to stay in treatment and on their medication. This engagement also suggests that clients may be willing to use a similar mobile health intervention to seek support for other clinical or logistical needs, hinting that future benefits are possible with this type of digital outreach. Combining cost and engagement benefits, the low-risk, high-reward of the 2wT approach would be a positive contribution to retention interventions in high-volume, low-resource settings. With the potential workload benefits of 2wT expansion [36,37] combined with the personalized, person-centered approach that aligns well with DSD goals of enhancing client engagement [38], the potential impact of 2wT expansion is high for both proactive retention and reactive client tracing efforts.

However, the rapid RCT also showed a lack of a significant impact on both ART retention and VLS at 6 months, in contrast with our earlier quasi-experimental findings that 2wT significantly improved ART retention 12 months after ART initiation. This discrepancy may be due to several factors. First, due to funding constraints, the pragmatic RCT follow-up was short (6 months vs 12 months), which may not have allowed time to observe direct effects on retention or VLS over time, including any potential mediation effect. Second, the quasi-experimental SoC clients started ART during the COVID-19 pandemic, when retention was lower overall. Third, the quasi-experimental study was conducted during the early stages of the EC program. Over time, the EC program may have improved and become more efficient. Lastly, in the quasi-experimental study, only the 2wT intervention group had verified mobile numbers, whereas the comparison group had a documented, but unverified, mobile numbers that could have belonged to others or not been operational. In contrast, all RCT participants had verified, operational mobile phone numbers at the time of the study, suggesting that these participants could have higher socio-economic or educational status. RCT participants, overall, may have been more likely to comply with visits regardless of intervention [10,39].

### Limitations

First, due to time and funding constraints, the analysis focused on a rapid results timeline. A longitudinal analysis with a follow-up time of 12–24 months may yield more robust findings. In addition, we did not account for treatment interruptions among clients who were LTFU and later returned to care, which may have led to a slight overestimation of follow-up time. However, given the short duration of follow-up, the number of clients affected is likely to have been small. Contextual differences from the quasi-experimental study may have led to an unrealistic effect size, resulting in a low power calculation. Additionally, the study was primarily powered to detect differences in retention, which may have limited our ability to detect significant differences in visit compliance or VLS. We could not confirm whether SoC received their intended support; SoC fidelity assessment was outside the scope of this small study. In line with national ART guidelines, baseline VL testing is not performed for new ART initiates and some clients were missed at 6 months; thus, we could not account for initial VL status nor include all individuals in our VLS analysis. Lastly, participation in the RCT was opt-in, so those who volunteered may have been more open to retention support. Despite these limitations, the results suggest that the lower-cost, lower-intensity 2wT improves ART refill visit compliance and maintains similar VLS and retention rates as SoC, making it a valuable tool to complement existing ART adherence and retention intervention.

### Conclusion

2wT improved ART refill appointment compliance within the first six months of ART initiation in a large public ART clinic in Malawi. Most participants actively engaged with the 2wT platform, and structured reminders successfully reached clients. Together, these results highlight 2wT’s potential to complement existing retention support and enhance early ART outcomes for those who are eligible and interested. However, 2wT did not significantly increase ART retention at 6 months and VLS. Additional impacts of 2wT may be delayed, warranting additional evaluation over time. Overall, if 2wT can improve ART refill visit compliance at low cost and high client engagement, with similar retention and VLS results, scaling 2wT alongside continued SoC efforts may have positive benefits for ART retention outcomes and service delivery efficiency in high-prevalence, low-resource settings.

## Supporting information

S1 Protocol Two-way Texting (2wT) to Improve Patient Retention While Reducing the Healthcare Workload in Two High-Burden Public HIV Clinics in Urban Lilongwe: a pragmatic randomized control trial (RCT). (PDF)

S1 Table CONSORT 2022-Outcomes Checklist. (DOCX)

S1 Fig CONSORT diagram Study participant flow. (DOCX)

S2 Fig Kaplan-Meier curve of retention on ART among 2wT and SoC clients over time. (DOCX)

S2 Table ART outcome of Participants Without Viral Load Measurement at 6 Months. (DOCX)

S3 Table 2wT Reminder Messages by Appointment Timing. (DOCX)s
