# Supplementary material for: Evaluating the effect of interactive two-way texting on 6-month antiretroviral therapy outcomes: Findings from a randomized controlled trial in Lilongwe, Malawi
Source: PLOS Glob Public Health. 2025 Sep 10;5(9):e0004598. doi: 10.1371/journal.pgph.0004598 (PMC12422422; doi:10.1371/journal.pgph.0004598)
Supplement: S1 Protocol — (PDF) [file pgph.0004598.s001.pdf]

Two-way Texting (2wT) to Improve Patient Retention While Reducing the Healthcare Workload in Two High-Burden Public HIV Clinics in Urban Lilongwe: a pragmatic randomized control trial (RCT)

Version 1.0

September 3, 2022

Collaborating Institutions:
Lighthouse Trust
Malawi Ministry of Health
University of Washington, Seattle WA USA

Funding Source:
National Institutes of Health, USA

Principal Investigator(s)/Primary Contact:
Jaqueline Huwa, Research manager, Lighthouse Trust

Hannock Tweya, PhD, MSc, Senior Evaluation Technical Advisor, I-TECH/Malawi
Caryl Feldacker, PhD, MPH, Assistant Professor, Department of Global Health, University of Washington, Seattle, USA

Table of Contents

Acronyms 3
Executive Summary 4
Participating Institutions and Roles 5
Investigators and roles 5
Funding Source and Mechanism: 7
Conflicts of Interest Statements: 7
Background 8
Justification 9
Study Objectives: 10
Broad objective: 10
Specific objectives 10
Methods: 10
Overall approach 10
Study design 10
Study setting and Population 11
Technology overview 11
Staff training 12
Sample size calculation 12
Study outcomes 12
Participant eligibility 12
Recruitment of 2wT clients: 13
Randomization 13
Routine ART initiation procedures (both arms) 13
Study procedures 14
Control/comparison group (Routine Back to Care (B2C)): 14
Data sources 16

Data analysis **16**
Retention: **16**
Limitations and uncertainties: 17
Protection of Human Subjects **17**
Consent Procedures: **17**
Risks to participants: 18
Protection Against Risk: 19
Confidentiality **21**
Ethics Training **22**
Potential Benefits of the Proposed Research to Research Participants and Others 22
Importance of the Knowledge to be Gained 22
Dissemination 23
Ghant chart of RCT research activities 23
Appendices: 24
Appendix 1: Client consent for routine 2wT, year 2 ..... **25**
Appendix 1a: Client consent for RCT **30**
References 35

Acronyms

2wT	Two-way texting
ART	Antiretroviral Therapy
ARV	Antiretroviral
B2C	Back to Care
DGH	Department of Global Health
DHA	Department of HIV and AIDS
EMRS	Electronic medical records system
HCW	Healthcare worker
HIV	Human Immunodeficiency Virus
HTS	HIV Testing Services
IEC	Information, Education, and Communication
I-TECH	International Training and Education Center for Health
IP	Implementing partner
LH	Lighthouse
LTFU	Loss to follow up
M&E	Monitoring and evaluation
MOH	Ministry of Health
MPC	Martin Preuss Centre
NHSRC	National Health Sciences Research Committee
PI	Principal Investigator
PLHIV	People Living With HIV
RTC	Randomized control trial
SSA	Sub-Saharan Africa
VL	Viral Load
VLS	Viral Load suppression
WHO	World Health Organization
UNAIDS	United Nations
UW	University of Washington

Executive Summary

In sub-Saharan Africa (SSA), retention of HIV-infection people in antiretroviral therapy (ART) care is an increasing challenge. With only 67% retained on ART in SSA, over 7.5 million people are out of care, threatening both individual health and HIV epidemic control. With significant healthcare system constraints and formidable healthcare worker (HCW) shortages, ART services in SSA struggle to meet ambitious global targets of 90% retained in care. In research settings, text-based mHealth innovations show promise to increase ART retention and, therefore, reduce viral load (VL). However, few mHealth innovations have been tested or proven effective in real-world settings with severe human and financial resource constraints. mHealth innovations that successfully retain more patients on ART within large-volume public ART clinics in SSA are urgently needed. Lighthouse Trust (LT), the largest public provider of ART in Malawi, operates two large clinics in Lilongwe with the Malawi Ministry of Health (MoH), Lighthouse Clinic (LH) and Martin Preuss Center (MPC), with a combined 35,000 ART patients. 12-month retention at Lighthouse is 67%, below the 90% global target for epidemic control. Both clinics employ a real-time electronic medical records system (EMRS) and implement a resource-intensive patient retention program, Back to Care (B2C). B2C aims to trace patients who miss ART visits by ≥14 days. MPC has over 7800 monthly ART visits, and more than 10% of clients are B2C-eligible. B2C demand, coupled with HCW constraints, results in tracing of only 33% of target clients. Lack of, or delayed, tracing reduces ART retention. Moreover, over 50% of traced B2C clients are found on ART, 60% of whom had transferred, demonstrating significant unnecessary tracing. Poor data quality reduces B2C effectiveness. Therefore, LT and the University of Washington's International Training and Education Center for Health (I-TECH), and Medic Mobile, implemented an innovative, proactive, patient retention system using two-way texting (2wT) between new ART patients and staff. A previous study (#20/06/2565 Two-way Texting (2wT) to Improve Patient Retention While Reducing the Healthcare Workload in High-Burden Public HIV Clinics in Urban Lilongwe) suggested that 2wT improved retention, resolved potential retention issues before they occured and improved data quality (e.g. identifying transfers), potentially reducing B2C workload. MoH requested a randomized control trial to create more rigorous evidence of 2wT impact. Therefore, with 2wT fully operational, we now implement a pragmatic randomized control at LT trial using routine 2wT teams. The pragmatic RCT will test the existing 2wT intervention to provide adherence support for, and visit-focused communication with, new ART initiates at both LH and MPC clinics, comparing 6-month retention (ART outcomes, visit adherence, VL) and B2C referrals among two groups of new ART clients: 2wT-supported (intervention) and routine B2C (control). With this rigorous evidence, we set the stage for national or regional 2wT scale-up.

Participating Institutions and Roles

Institution	Roles
Lighthouse	• Lead the development of the study design<br>• Implement study activities in collaboration with the Ministry of Health, relevant technical working groups and stakeholders<br>• Conduct daily supervision of project staff and ongoing support at participating sites<br>• Conduct necessary training to implement this project<br>• Co-lead data management, analyses and publication<br>• Provide oversight of communications, reporting, and staff
University of Washington	• Secure funding, serving as administrative and financial oversight<br>• Lead study conceptualization<br>• Co-lead project design and protocol development<br>• Review and approve the protocol<br>• Co-lead data analysis and publication<br>• Lead all reporting to the NIH<br>• Provide technical assistance with regards to study design, international IRB and local IRB approvals, implementation, evaluation, publication
Ministry of Health	• Provide inputs to implementation, analyses and publication<br>• Support integration of the project system into routine EMRS<br>• Provide insight to policy needs and implications
Medic	• Development, implementation, and supervision of the technology for two-way texting<br>• Responsible for ensuring open-source software is integrated into EMRS according to research timeline<br>• Training for technology for Lighthouse teams

Investigators and roles

Name	Title/affiliation	Role on project	Contact details
Hannock Tweya, PhD, MSc	Monitoring, Evaluation and Research Director, Lighthouse Trust	Co-Principle Investigator<br>• Provides overall direction and oversight for project management and implementation at Lighthouse<br>• Oversight for program implementation according to approved protocols	htweya@lighthouse.org.mw

		• Provides assistance to Project Team, as needed, in the development of study design and methods, implementation, evaluation, and publication.<br>• Responsible for technology coordination and system implementation oversight	
Caryl Feldacker, PhD, MPH	Assistant Professor, University of Washington, Seattle, WA, USA	Co-Principle Investigator<br>• Responsible for NIH reporting and overall research oversight<br>• Provides technical assistance to Project Team, as needed, in the development of study design and methods, implementation, evaluation, and publication.<br>• Provides supervision and mentoring to junior staff involved in the project<br>• Coordinates and supervises the work of technology partner, Medic Mobile	cfeld@uw.edu
Jacqueline Huwa	Research manager, Lighthouse Trust	Co-Investigator<br>• Provides supervision for project management and implementation at Lighthouse<br>• Provides assistance to project team in the development of study protocols including SOPs<br>Fosters and facilitates communications with collaborators and stakeholders	jhuwa@lighthouse.org.mw
Beatrice Wasunna, PhD	Medic Mobile, Nairobi Kenya	Investigator<br>• Leads and supervises all aspects of technology design, development, implementation and integration.<br>• Provides quality assurance and supervision for the technal Medic Mobile team for project implementation.	isaac@medicmobile.org
Rose Nyirenda, MPH	Director, HIV Department, Malawi Ministry of Health	Investigator<br>• Provides insight to policy needs and implications<br>• Provides assistance to Project Team, as needed, in the development of	rnirenda@hivmw.org

		study design and methods, implementation, evaluation, and publication.	
Thom Chaweza, DCM, MPH	Medical Officer, Lighthouse Trust	Investigator<br>• Responsible for project coordination and monitoring<br>• Provides assistance to Project Team, as needed, in the development of study design and methods, implementation, evaluation, and publication	tchaweza@lighthouse.org.mw

Funding Source and Mechanism: National Institute of Health 1 4R33TW011658-03 entitled Two-way Texting (2wT) to Improve Patient Retention While Reducing the Healthcare Workload in High-Burden Public HIV Clinics in Malawi.

Conflicts of Interest Statements: No conflicts of interest to declare.

Background

In Malawi, with an estimated 1 million (9%) living with HIV [1], 5-year ART retention falls to 54% [2], stymying progress towards HIV epidemic control [3, 4]. **Reactive retention efforts are resource intensive**, reducing likelihood of scale or sustainably [2]. Lighthouse Trust (LT), a national ART Centre of Excellence in Lilongwe, Malawi, operates with the Malawi Ministry of Health (MoH) to provide HIV care, treatment, and support across Malawi. In its two Lilongwe flagship clinics, 35,000 patients are on ART: 24,000 at Martin Preuse Centre (MPC) and 11,000 at Lighthouse (LH). Patients at both are managed via a real-time, point-of-care, electronic medical records system (EMRS) [5-9]. Despite its prominence, retention at MPC and LH clinics falls short: an average of 67% of ART patients are retained at 12-months, well below the 90% threshold needed for epidemic control [10]. Since 2006, LH and MPC implemented a patient retention program, "Back-To-Care" (B2C), that traces ART patients by phone or home visit who miss a clinic visit by >14 days. B2C has been well-recognized for its success returning patients to care [11-18]. Although early patient tracing increases likelihood of patient reengagement [19], tracing-based retention efforts are reactive -- waiting for clients to miss visits before intervention. B2C and other tracing programs are highly resource intensive [20], limiting scale. At MPC, there are over 7800 scheduled ART visits each month: of these, over 800 clients are presumed LTFU and referred to the 8 full-time B2C tracers. Program resource constraints cause B2C delays and tracing gaps, greatly diminishing B2C effectiveness and retention success. **In December, 2019 only 30% of eligible clients were traced at MPC**, a tracing gap found in other ART programs in SSA [21-23]. Additional proactive retention efforts, within the existing limited resources, are needed to reduce LTFU before it happens.

Myriad mHealth interventions, including text-based, improve ART retention in research settings. However, evidence of successful transfer of these gains to routine ART services delivery in low-resource settings and at scale are missing [24, 25]. With expanding internet access [26], mobile phones are common tools for healthcare needs, even in rural areas [27]. As such, the World Health Organization (WHO) promotes mHealth interventions in low resource settings to support ART adherence [28]. These interventions appear highly acceptable to clients [23, 29-32]. Although mHealth retention efforts aimed at youth [33`] or implemented within already-high retention settings (over 80%) may not be effective [34, 35], numerous text-based research interventions significantly increased ART retention (adherence, visit attendance, and viral suppression) among adults [36-45]. Digital innovations also increased engagement among men [23, 38, 46, 47] who are often poor users of health services [48-51]. Interventions that "blast" one-way pre-defined, messages to many people, simultaneously, increase ART adherence [22, 42, 52]. **Interactive, two-way texting (2WT) improves upon blast messaging by encouraging direct client to HCW communication, increasing engagement in care [53] and improving care outcomes** [43, 44, 54, 55]. However, some 2WT intervention require intensive HCW effort, limiting scale-up [44, 56]. mHealth innovations need to provide workload advantages for frontline HCWs who must manage these efforts [54, 57-59].

We successfully led a 2WT-based randomized control trial (RCT) among male circumcision (MC) clients in a routine MC clinic in Zimbabwe. We improved the quality of care and reduced provider workload by 85% [59] **while reducing costs by 33%.** Our 2WT extended the reach of HCWs to effectively monitor more patients while improving the quality and

timeliness of routine data. Both clients and HCWs found the system highly usable and acceptable: 93% of men responded to texts [60]. The routine messages and real-time provider feedback engaged clients as care partners. Similarly, providers received real-time information from clients, allowing for timely reporting of routine data. This project will build on successful 2WT technology developed in Zimbabwe by this team, adapting it for retention efforts in high-burden, public ART programs in Malawi. **Engaging patients in retention efforts could similarly improve client care, data quality and HCW workload at lower cost**.

Lighthouse and its B2C program appear primed for this new kind of task-shifting where mHealth tools enable clients and care givers to exchange information directly and quickly. The hybrid workflow designed for the Zimbabwe RCT integrated the efficiency benefits of automated messaging with the familiarity of SMS on basic phones common in Zimbabwe and Malawi. The intervention also implemented core characteristics of mHealth excellence, including optimizing client accessibility and acceptance; low technology costs, local adaptation, strong stakeholder collaboration, and government partnership for sustained impact [61]. With the same fundamental approach, **our experience suggests ART retention efforts and B2C workload would benefit similarly** because: 1) tracing is burdensome to providers; 2) the intervention enhances routine care quality, minimizing risk; 3) clients can remotely update outcomes in real-time (transfer, travel, other), improving data quality; and 4) frequent visits reduce issues like lost phones, new numbers, etc. [30]. 2wT was successfully tested in 2020-2022 with initial results suggestive of modest increases retention among 2wT participants with high uability and acceptability among LT staff.

The current 2WT improves upon previous texting interventions for retention in several significant ways: 1) Hybrid 2WT with a mix of automated and human-to-human messaging is optimized for staff efficiency by proactively and directly engaging with clients, preventing default (changing visit dates) or ascertaining true outcomes (transfer, stop, etc.); 2) Real-time data provided by clients will improve ART outcome data before 14 days, reducing unnecessary referrals to B2C for tracingand 3) Medic Mobile's open-source intervention software will aid efforts to adapt, maintain, and sustain the system, leveraging the global community of developers to contribute to design improvements in inbox management, message prioritization, or task management.

Justification

Two-way texting, itself, is not novel for retention. However, we developed and evaluated a low-cost 2WT intervention to increase ART retention within a high-volume, routine, public healthcare setting. 2wT is timely, innovative, and programmatically important, aiming to close the gap between what works in research and in routine practice. Lighthouse's hybrid 2wT system among new ART clients at MPC clinic, the largest single clinic for ART patients in Malawi is designed, developed, and initially evaluated with preliminary results appearing positive. The previous study is nearing completion under approved protocol: #20/06/2565 Two-way Texting (2wT) to Improve Patient Retention While Reducing the Healthcare Workload in High-Burden Public HIV Clinics in Urban Lilongwe). This complementary, small-scale, pragmatic randomized control trial (RCT) will strengthen results to inform scale-up while subsequent expansion into routine B2C operatations allows for routine evaluation of 2wT in practice. The RCT will provide rigorous evidence for future expansion by implementing 2wT within both LH's and MPC's

existing, public ART program. Comprehensively evaluating 2WT's impact on patient retention and B2C referrals under routine pragmatic conditions will provide real-world evidence of its value, greatly improving the relevance of findings and likelihood of program expansion. Using the RCT results will further optimize 2wT for expansion in routine B2C efforts, supporting LT's retention program. See key innovations: Appendix 6, Table 1.

Study Objectives:

Broad objective:

To determine if the innovative two-way testing mHealth intervention can significantly increase retention on ART among PLHIV, reducing patient tracing workload.

Specific objectives

1. To determine how much 2-way, visit reminder texting between the Lighthouse clinic and Lighthouse patients can increase new ART client retention and reduce the tracing workload within the first 6 months on ART

Methods:

Overall approach

Overview (See key activities and study timing in Appendix 7, Table 2): Building on the previous 2wT study, this study conducts an RCT for strengthened evidence generation. Over 12 months, we will conduct an RCT in both MPC and LH to prove if 2WT increases 6-month retention (clinic visits, ART outcomes, VL) and reduces workload in both MPC and LH clinics. 6-month outcomes are used as the timeframe is short due to funding constraints. For the RCT, no changes are required in the 2wT software beyond a field for randomization arm. No changes to the educational materials nor to the implementation are needed. The RCT will enroll 410 participants, randomized 1:1 2wT and control. At 6 months, all participants will receive 2wT and continue with 2wT in routine retention activities. The RCT will provide rigorous evidence and generate lessons in direct response to the request of the MoH DHA for more evidence of 2wT impact.

Study design

In this current pragmatic RCT study, we randomize eligible 2wT patients who wish to opt-into 2wT, allocating them 1:1 to 2wT and routine Back to Care (B2C) retention efforts. For 6 months, each participant will receive the assisged intervention (2wT or control). At 6 months post enrollment, all RCT participants will receive 2wT and continue with 2wT support as part of routine LT retention activities. We will enroll at both LH and MPC, using results to provide the MoH with data needed to consider 2wT for scale-up.

Study setting and Population

MPC and LH clinics are in urban Lilongwe, largest public clinic that currently serve over 25,000 patients alive on ART. About 60% of patients are female. On average, MPC and LH clinic initiates **over 800 clients on ART per quarter**, ensuring sufficient pace of enrollment.

Technology overview

Since 2008, Medic Mobile has been a leader in the global mHealth community [62-68], equipping more than 26,000 health workers serving over 12 million people across 25 countries. Medic Mobile is the steward of the Community Health Toolkit (CHT), an open source project (https://communityhealthtoolkit.org/) supporting dozens of mHealth implementations, including 2WT in Zimbabwe. Interventions and apps built using the CHT work with or without internet connectivity, in any language, on basic phones, smartphones, tablets, and computers [69] and are tailored to meet key digital health characteristics recommended by the World Health Organization (WHO) [70]. The core areas of functionality used by the current 2WT system include peer-to-peer and automated interactive messaging, task management features (unread message; message not delivered), longitudinal patient records, data collection forms, routine syncing, and dashboard reports for routine monitoring (e.g. client response rates, referrals, etc.) [58]. Together, these features will enable an integrated 2WT workflow tailored to ART retention, generating the data needed to monitor program delivery and facilitate research. The CHT also supports interoperability with other eHealth tools (via the NIH-supported FHIR standard [71] or custom data formats). The existing 2wT flow diagram is illustrated below.

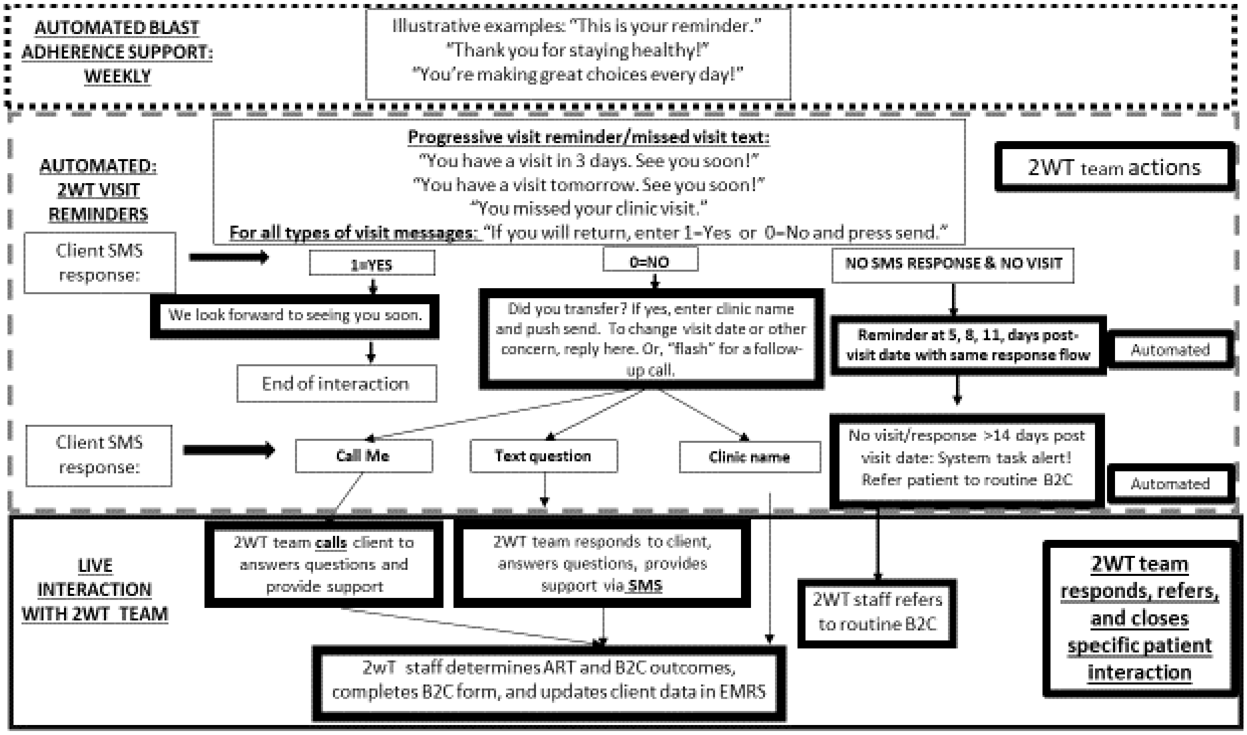

Staff training

All staff at LH and MPC will be informed of the study and briefed on study protocols. A 2WT study coordinator, selected from current staff, will be trained in confidentiality, protection of human subjects, enrollment, randomization, and data collection methods. This person will manage enrollments, randomization, and the 2WT database, including text and voice follow-up communication with intervention clients. Malawi-based co-investigators, including MoH collaborators, will also be invited for Medic Mobile training.

Sample size calculation

Supposing a two-arm prospective study with 3-4 months recruitment period and 6 months follow-up. Assume the hazard for the control group is a constant risk over time at 0.2 (20%). To achieve 80% power to detect a hazard ratio of 0.41 in the exposed group by using a two-sided 0.05-level log-rank test, the required sample size per arm is 197. With an estimated dropout of 10%, 410 (205 per arm) will be needed, and up to 500 will be enrolled, total, to allow for buffer and withdrawal of those with lack of SMS confirmation receipt (documented).).

Study outcomes

Primary retention outcomes: 1) Proportion of patients retained in care at 6-months; (2) visit attendance; and 3) VL (suppressed = HIV RNA < 400 copies). Primary workload outcome is cumulative B2C referrals at 6 months. Secondary outcomes include: Text response rates, #visit date change requests, #ART outcomes updated, % of failed messages, client contact number updates, % referral of true LTFU for tracing, and B2C tracing outcomes (transfer, stop, alive, etc.).

**Table: Outcomes**	Definition	Source	Phase	
**Individual level (Reach and Effectiveness):**			**R21**	**R33**
**Retention outcomes**				
Retention	6-month ART retention (alive on ART or client report of transferred care)	EMRS	X	X
ART visit adherence	% of visits attended		X	X
Viral load	% suppressed (HIV RNA <400 copies)			
**Workload outcome:**				
Healthcare workload	# cumulative B2C referrals at 6 months; time spent on 2WT and B2C	Time-motion, EMRS, 2WT database	X	X

Participant eligibility

The intervention will be offered to all new ART registrants and new ART initiates (clients who initiated ART in the last 6 months) at MPC and LH aged 18 years and above with mobile phones. This allows for collection of all outcome data by 12 months. 2WT-specific eligibility criteria for all clients are: 1) New ART registrants (Initial screening visit for ART initiation) OR new ART initiate (within 6 months of initiating ART); 2) ≥18 years; 3) possession of own phone at enrollment; 4) willing to receive/send SMS; 5) informed consent; 6) receives confirmed 2WT enrollment text to verify enrollment number; and 7) initiates ART. Those without cell phones will

be excluded. As phone sharing is common, informed 2WT-specific consent will ascertain whether eligible clients have consistent access to send and receive messages. Patients will see example text context that will not contain HIV- or clinic-based information to reduce risk of disclosure; these steps have been used in similar SMS-based studies [72, 73]. 2WT cannot control message content sent by clients. Study-eligible clients will be automatically enrolled into 2wT messages with confirmed ART dispensing. Consenting 2wT enrollees who do not initiate ART will not be sent messages.

Recruitment of 2wT clients:

Information about the study will be disseminated through MPC teams. As the backbone of the intervention, the Back to Care program is already part of the core patient counseling for ART registrants, taking place before ART initiation. Adding the study-specific information will occur during this routine part of ART initiation counseling, while clients are considered *ART registrants*. Over 92% of ART registrants initiate ART the same day. Recruitment will be managed by a specifically-trained research coordinator who will be a part of the Back to Care program. This research coordinator will meet with incoming ART initiation patients to sensitize them about the opportunity to participate in a study of text-based follow-up; those meeting eligibility criteria will be informed as a group about the opportunity to participate in the study. Flyers will also be available in clinic waiting areas and counseling rooms. Interested patients at ART initiation visit or within 6 months of ART initiation will be referred to the study staff for study enrollment and informed consent. Participant enrollment may be made by teams/clinicians using the Medic Mobile phone/tablet app or the laptop-based system.

Randomization

After routine recruitment consistent with 2wT procedures, eligibility, and informed consent, 410 2wT-eligible participants will be randomized 1:1 intervention and control using a randomized block design in groups of 10. Assignments will be held in opaque envelopes distributed to LH and MPC to meet enrollment pace. This block design reduces variability within treatment conditions and potential confounding, allowing near equal-size groups for interim analysis. Site coordinator will be unaware of the envelope's contents until the participant opened and revealed the group assignment to the coordinator. Coordinator then enters participant group assignment as part of 2wT enrolment, confirming phone number as is routine. Those who do not receive the confirmation text while in clinic will be immediately withdrawn and replaced. The study design requires that participants and clinic staff are aware of allocation after assignment as routine early retention and B2C processes differ from 2wT.

Routine ART initiation procedures (both arms)

All ART care is provided free to clients as mandated by MoH. All diagnosed HIV-positive individuals who report to clinic reception are registered in the EMRS (ART registrants) and initiate ART same day (ART initiates). All new registrants receive augmented adherence counseling as part of the ART initiation visit. New ART patient visits are scheduled monthly during the first six months on ART and every two or three months thereafter if the patient is stable and adherent to therapy. Routine VL testing occurs at initiation and annually, thereafter. Clinic visits, side effects

and dispensed drugs are captured in EMRS in real-time by clinic staff. LH and MPC follow all MoH protocols based on WHO guidelines [74] including standard indicator reporting: ART outcomes (dead; transferred; alive on ART; stopped ART; never started ART; and LTFU), VL, and adherence [75].

Study procedures

Processes between 2wT and routine Back to Care (B2C)

Table Key activities overview	Increased retention via Two-way texting	
**Specific Activities**	Control	**2wT**
**PROACTIVE ART RETENTION**		
Automated blast weekly adherence SMS for new ART initiates		X
2wT hybrid visit reminders 3 and 1 day before visits		X
2wT interaction/calls for those respond "no" to visits		X
**EARLY ACTIONS TO REDUCE LTFU AMONG 2WT ENROLLEES**		
2wT hybrid visit reminders at 5, 8, 11 days post visit		X
Updating of client reported ART outcomes (transfers, etc.) <14 days		X
If no client visit/contact, day 14 alert created: refer to B2C		X
B2C phone and home tracing of patients presumed LTFU	X	X

Control/comparison group (Routine Back to Care (B2C)):

All patients are eligible for B2C. Patients with any ART drug dispensing record (including initiation) who missed a scheduled appointment by 14 days or more are identified using the EMRS and considered LTFU. A team of B2C tracers manually verifies the LTFU list to rule out EMRS data errors and creates a paper-based B2C tracing list. Then, patients with completed locator forms are first traced by phone (SMS or call) up to five phone attempts and/or then up to three home visit attempts. The EMRS at ART patient registration is used daily to determine if/when a client returns to care and to stop B2C client follow-up for that specific event/month. For all patients reached, B2C interviews clients to ascertain ART outcomes and records data on paper B2C forms; data clerks enter it into the EMRS.

2WT (intervention):

All new ART registrants who fill locator forms will be asked to consent to 2WT; those who enroll will receive an enrollment confirmation text, additional texting information, and response instruction at initiation. ART initiates who fail to bring their phone at their first visit or otherwise decide to participate after the initiation visit will be eligible to join the study at any visit within the first 6 months on ART. They will also be asked to consent to 2WT; those who enroll will receive an enrollment confirmation text, additional texting information, and response instruction at enrollment. 2wT paper clients files will be marked to alert reception of participation. Then, 2WT data clerk will manually enter client unique ID, client phone number and scheduled visit dates into 2WT system. Successful SMS receipt is verified by 2WT's system; failed SMS creates a system report. Based on these inputs, the 2WT system will automatically send varying, weekly adherence

support texts and visit reminders (3 and 1 day before) for 12 months, with opt-out at any time. Clients will be asked to respond to visit reminder messages with a "1=will attend" or "0=no." A "yes" will trigger, "See you soon!" A "no" will trigger hybrid automated and interactive SMS follow-up where clients can request an appointment date change, report transfers, note other outcome, or request a call. These modifiable, scripted texts between 2wT staff and the client will complete ART outcome data (transfer, stop, etc.) or determine the next steps (**Figure 2** for mock-up).

To support visit verification, the 2wT system will produce a set of daily "tasks" (IDs of client with expected visits) for 2WT clerks to monitor visit attendance. Using these daily lists of presumed visits, a clerk will confirm visits using the EMRS, recording attended visits in the 2WT system, updating subsequent visit dates if needed. Confirmed attendance stops visit reminders for that specific instance; completion of other ART outcomes ascertained via 2WT (stop, transfer, dead) also stops all messaging. If an expected client visit was not confirmed, and outcome otherwise not updated, the 2wT system will create another alert after 5 days, triggering 2wT staff to launch an automated a series of "missed visit" SMS with similar 2WT response options every 3 days until day 14. If no visit, no text, or otherwise updated ART outcome by day 14, the 2wT system will create another alert for referral to routine B2C. B2C referrals will be compiled weekly. The 2WT-based generation of the B2C list with reported transfers removed and visits verified, will reduce delays in identification and tracing of those presumed LTFU. **The system is free for clients.** See text message flow: Appendix 11, Figure 2 and Appendix 12: 2wT interface mock-up.

Specifically, procedures for 2WT RCT participants will include the following:

1. Patients will have routine Lighthouse adherence counseling as per new ART initiates
2. For those eligible, desirous to opt-into 2wT, and who consent, they will be randomized 1:1 into the 2wT retention or routine retention groups
3. In addition to routine care instructions, 2WT clients will receive additional counseling on the texting system and expected responses, and instruction in how to use the texting system before enrollment at either ART registration or within 6 monts of initiating ART.
4. For follow-up, 2WT clients will receive automated weekly texts from initiation through 12 months. They will also receive message 1- and 3-days before scheduled visits. Flow chart of texting is included in Figure 2. Automated texts, as illustrated in Science, will be in either both English or Chichewa, as per client selection at enrollment. It is free to use the system for clients.
5. If they respond that they will attend a visit, no immediate follow-up action will be taken.
6. If a 2WT ART client responds they will not attend a visit, a 2WT staff will exchange modifiable, scripted texts with the client to determine to change visit date, record transfer or other. Multiple responses and free text are acceptable. Regardless of response, the 2wT will enter into interactive texting with the client.
7. No client has to respond to messages
8. Any 2wT client, as per routine care who does not respond and does not attend any visit by >14 days will be referred to routine B2C

9. Data from the 2wT toolkit, participating clients, and ART forms will be reconciled by the Lighthouse study team as per routine monthly reporting requirements. Data on all visits will be entered into the online 2wT toolkit database.
10. Brief, self-administered, mixed-method surveys on 2WT usability (Appendix 3: usability survey with clients) will be implemented at ART visits at 3- and 6-months with a subset of 100 2WT clients (50 at 3 months/50 at 6 months) to gauge client acceptability, self-efficacy, perceived benefits, and barriers to 2WT for intervention improvement. Clients may refuse to participate in these surveys and will be replaced to reach 100.
11. At 6 months, RCT participants who were randomized to routine retention will be enrolled into 2wT

Data sources

During the course of the study, in both year 1 and 2, study team members will review existing paper-based records at the site with identified patient data, including paper ART visit forms for purposes of data quality assurance. Data will be collected across two objectives: data abstractions from participants'routine ART data in the electronic medical records system (EMRS) as well as supplemental information from the 2WT database. EMRS data is routine data. 2WT system data entry and forms will be checked for completeness and correctness on a weekly basis. Data for analysis will be abstracted only from the study database.

Data abstracted from EMRS:
- Basic demographic data such as name and age, WHO stage
- Routine ART visit dates, viral load, adherence, ART outcomes

Supplemental, study-specific data includes:
- Enrollment form: confirmation of study consent, enrollment date, phone company, cell number
- Non-routine data in 2WT database: client messages exchanged with 2WT staff, message failures, visit date changes, ART outcomes completed.

<u>Data for program retention</u> (clinic visits, ART outcomes, VL), will be extracted from routine EMRS for both 2WT and comparison clients. B2C referrals and outcomes will come 2WT database (intervention) and from the <u>routine B2C database</u> (comparison). Process data from the <u>2wT database</u> includes all other data for secondary outcomes.

<u>Data collection timing:</u> prospective for new initiates in 2WT and retrospective comparison without 2WT.

Data analysis

Retention:

Descriptive statistics will be performed to explore the patients' characteristics at ART initiation using means or medians for continuous variables and proportions for categorical variables. Chi-square test and Kruskal-Wallis tests will be used to compare baseline characteristics. The proportion of patients retained in ART care (or known transferred in care) at 6 and 12 months post-ART initiation between groups using chi-square test will be compared, reporting relative risks and 95% confidence intervals (CIs). Time to event analysis will explore retention curves between groups in the first year of ART. T-test will compare the mean proportion of attended ART visits between groups. VL suppression (HIV RNA < 400 copies) at 12 months post-ART initiation will be compared between groups using chi-square test, reporting relative risks and 95% CIs. Multivariate logistic regression models (12-month retention v not) will quantify the magnitude of retention difference between groups, adjusting for any potential confounders (age, sex, WHO stage at ART initiation, etc.). Statistical analysis: B2C referrals (workload) outcomes: T-test will compare the cumulative number of B2C referrals between groups. A multivariate linear regression model will further quantify the effect of intervention on referrals, adjusting for potential confounders. For the RCT, the above outcomes will be measured and compated between arms at 6 months.

Limitations and uncertainties:

While the study retention target of 82% still falls short of the global goal of 90% retained, it is well above the global average of 67% and acknowledges that improving retention is a multipronged approach. Also, a 15% increase in retention is ambitious for a routine, public, urban setting and would translate to a 55% reduction in B2C tracing workload. As costing analysis takes a conservative approach, looking at direct costs and not accounting for potential benefits accruing from avoidance of morbidity and mortality [76], sensitivity analysis may reveal that the break-even point for 2WT cost advantage over routine care is even lower than15%, requiring a smaller increase in retention to be cost-effective. Phone ownership in urban Malawi is high, but if the clinic population ownership is lower, the effect of 2WT will decrease. It is possible that clients will not respond to SMS, increasing the need for routine B2C, thus reducing the efficiency and cost gains afforded by 2WT.

Protection of Human Subjects:

Consent Procedures:

In obtaining informed consent, the study member enrolling patients will follow a strict script that emphasizes: 1) participation in the study is completely voluntary; 2) whether or not they participate will in no way adversely affect they care they receive. Consent procedures will occur before enrollment in the intervention study.

The consent documentation was written by the Malawian staff and reflects the average literacy level of their clients. Study staff will be available to answer any questions. The informed consent process is initiated prior to an individual agreeing to enroll in the cohort and continues throughout

the individual's participation in the cohort. Patients who choose to enroll in the study will be provided with the enrollment informed consent, which describes in detail the study procedures and risks. Upon reviewing the document, the site staff member explains the study to the subject and answers any questions that may arise. Discussion of risks and possible benefits of study participation will be provided. The participant signs the informed consent document prior to the conduct of any study procedures. A copy of the enrollment consent form is given to the subjects for their records. Participants, both clients and providers, will also be informed that they can withdraw from the study at any time with no repercussions.

This RCT is registered with ClinicalTrials.gov (ClinicalTrials.gov Identifier: NCT05531448) and will receive UW approval as an amendment to current 2wT IRB approvals before implementation. Current consent documentation is updated with RCT-specific requirements, attached.

Patients (Appendix 1: Client consent): Interested patients will be referred to the site 2WT study coordinator for study enrollment and informed consent. This person will meet with patients in the ART initiation counseling room (where clients are considered *ART registrants*), further explain the study, confirm study eligibility, and seek informed consent. For clients within the first 6 months of initiation who are interested in 2wT, they will also meet with the 2wT coordinator in the counseling room to further explain the study, confirm study eligibility, and seek informed consent. Participant enrollment may be made by teams/clinicians using the Medic Mobile phone/tablet app or the laptop-based system. Subject recruitment and consent will be in English and Chichewa. All staff are fluent in both English and local languages for translation and transcription of activity materials. Consent forms, advertisements, and study information materials will be translated by the local study team.

Risks to participants:

Injury/side effects/adverse events: All routine ART care procedures will be followed for this study. Any additional risk that may be incurred by participants by virtue of their text-based follow-up will be monitored closely and managed as described above. Participants will also be informed that they can withdraw from the study at any time with no repercussions.

Discomfort/stress: Individuals who consent to participate may experience mild discomfort or stress as they will be asked to discuss topics that may be sensitive, including their opinions on the intervention. We will prevent undue influence by stressing the voluntary nature of this study and each person's ability to decline without any risk to their ART service quality.

Specific for participants, this study carries some small risk of social harms for clients. ART clients face a risk of stigma from HIV as their HIV status may be inadvertently revealed to others outside the study as a result of their participation or as a result of the texting intervention. ART clients who participate in surveys may feel uncomfortable answering questions about service quality and their opinions about the intervention.

Protection Against Risk:

1. Procedures for minimizing risk to participants include the careful selection, training, and supervision of study personnel and site-level study liaisons, together with the systematic preparation of other HCW involved in study operations. Study personnel are selected for past training and ability to work with participants in this target population. All site-based study personnel (project site coordinator, data clerk) will be working specifically for this study from partner organization, Lighthouse, and trained in procedures to ensure confidentiality in research settings. They will also be trained in research ethics and human subjects' issues via the Collaborative Institutional Training Initiative (CITI) training. At each site, there will be a site-level study liaison who is a regular staff member at the health facility who will be informed on study recruitment procedures, confidentiality protection, and documenting study operations. Dr. Feldacker will also take the Clinical Research Training On-Line Course for Principal Investigators and any additional courses suggested by University of Washington Or NIH to further her understanding of ethical conduct of clinical research.

2. The study database with study-specific data will include only coded information and no names or identifying numbers which could be used for individual identification of participants. Safeguarding confidentiality of personal data reported on study surveys and questionnaires will be achieved through identifier codes instead of full names on all research materials. The list referencing code number to name will be kept in a locked file cabinet in the research manager's private office and coded data will be stored in computers in locked offices to which only project staff have access. The list relating names to number codes will be destroyed at the end of the study. Secondary data containing identifying information will be used to match records for patients who specifically consent to use of their health records, between the routine ART data and the study-specific database. Following the matching of data sing names and codes, the identifying information within the dataset abstracted from the secondary data sources will be destroyed and only the study-specific coded identifiers will be preserved. No individually identifiable information from ART clients will be published.

3. Safety and Progress reviews:

1. ART outcome among all Lighthouse/MPC clients, including those 2WT, will be reviewed quarterly as part of routine care. LTFU will be reviewed quarterly as per routine care.
2. Annual report will include:
    - Progress report: summary of recruitment and retention and reason for dropouts;
    - whether LTFU are consistent with pre-study assumptions;
    - whether the study is on track to be completed and accomplish the stated objectives.
    - Technology challenges
    - Study challenges
    - Success and preliminary data

4. Linkages to subjects and access to subject identities: LH and MPC use unique IDS that will be used in this study to ease processes and data linkages. Routine EMRS data practices that safeguard

this data as part of rouinte EMRS use will be employed in addition to restricting the 2WT database access to only the study team. Confidentiality will be maintained by assigning a unique study-specific ID for all non EMRS data collected as part of the study.

DATA QUALITY AND MANAGEMENT

Quality Control will be ensured through the timely review of all study data to verify accuracy and validity. QC will take place on a weekly basis, allowing for swift review and correction if needed. Validation review will also happen weekly. The 2wT study database with study-specific data will include only coded information and no names. Safeguarding confidentiality of personal data reported on study surveys and questionnaires will be achieved through identifier codes instead of full names on all research materials. The list referencing code number to name will be kept in a locked file cabinet in the research manager's private office and coded data will be stored in computers in locked offices to which only project staff have access. The list relating names to number codes will be destroyed at the end of the study. Secondary data containing identifying information will be used to match records for patients who specifically consent to use of their health records, between the routine VMMC data and the study-specific database. Following the matching of data, the identifying information within the dataset abstracted from the secondary data sources will be destroyed and only the study-specific numeric identifiers will be preserved. No individually identifiable information from ART clients will be published. Databases will be password protected and shared through encrypted file sharing services.

Medic Mobile Data Security

All data in the Medic Mobile database is stored on users' local computers, and then synced to a cloud server hosted by Amazon Web Services. We make use of Amazon's developer tooling to automate the process of creating routine back ups of all data that reaches the server. This means that data can only be lost if a health worker is using the software on a computer that is fully offline, and then the computer is damaged/stolen before connecting to the internet. In this scenario, the project would only lose data generated since the computer's last sync to the server. The Excel study database will be backed up on a Google cloud server via the Google Apps service. Both of these cloud services are widely used in health services in the United States and around the world, in part because they provide very high levels of data security, and back-up and other data management tools.

Medic Mobile is committed to data security and follows the data protection standards set by the Ministry of Health or our in-country implementing partner, as appropriate. We recognize the security benefits and drawbacks of different technology tools, and work with our partners to make the best choices and mitigate risk.

- Web app: Medic Mobile uses secure transfers over HTTPS for all communication between the browser and our web application, with perfect forward secrecy (PFS) and 4096-bit SHA-2 certificates by default. We use a non-standard port for SSH access to reduce our exposure to automated brute-force attacks, and can configure the web app to accept only public key

authentication for SSH connections. Access to the web application requires a password, and user access can be established to varying degrees using a role-based access control facility (e.g. full access, restricted access, data entry only, and data export only).

• Data storage: Medic Mobile uses Amazon Web Services (AWS) with enforced two-factor authentication, HTTPS, and Identity and Access Management (IAM) for all hosted instances. We use IAM policies on AWS to restrict what any one individual Medic Mobile developer/administrator can do. The data storage system is backed up daily.

• SMS: We train users to input data using simple SMS codes or freeform SMS. We use

"plain text encoding" which means viewers can see the value but not know the context of the data. SMS is inherently insecure but we work with every partner on safety practices to reduce and minimize mishandling of data and transmission of protected health information.

• Android phones: Full disk encryption with an auto-lock screen will be deployed.

Security training for our partners: Staff will trained and equipped to create secure passwords and PINs, secure hardware, and how to safely transport data.

Confidentiality

Patients' routine ART data from EMRS, including patient demographics, clinical characteristics at baseline and during follow-up period. This data will be entered into the study-specific database. For the study specific database for analysis, IDs and no names will be used, as is routine with Lighthouse research. Confidentiality will be maintained by assigning a unique study-specific identifying alphanumeric code to each participant's data in the 2WT database.

Encryption will also be used wherever data are transferred. Implementation strategies for data management will take full consideration of regulations on confidentiality and security of electronic data transmission and storage. Upon completion of this study and exhaustion of the data retention period, all study identifiers will be deleted. In addition to the study staff's maintenance of confidentiality, healthcare workers involved in the study will be oriented to the importance of confidentiality and their own roles in maintaining confidentiality.

Data containing identifying information will be used to match records for patients using the EMRS and the study-specific database. Following the matching of data sing names and codes, the identifying information within the dataset abstracted from the secondary data sources will be destroyed and only the study-specific coded identifiers will be preserved. No individually identifiable information from ART clients will be published. All members of the study team (including Medic Mobile) that interact with data sign a confidentiality agreement. Sensitive data is de-identified and/or made inaccessible to certain employees. Medic Mobile's protected health information (PHI) policy requires that developers, employees, and contractors use full-disk encryption on any computers receiving or manipulating PHI.

Ethics Training

All staff who have contact with participants will receive training on the protection of human research participants and Good Clinical Practice (GCP) prior to conducting any study activities and every three years thereafter. Lighthouse Trust will facilitate ethics training for staff during the award. We will seek appropriate approvals from Institutional Review Board (IRB) at the University of Washington (UW) as well as the National Health Sciences Research Committee before contacting any human subjects to ensure that the study complies with both UW and local laws and requirements. Because this project involves local health authorities and we have successfully received UW and Malawi-based ethical review for other health-related research projects in Malawi, we do not expect any problems or delays with this approval. Below we discuss our plan to minimize risks to human subjects involved in this research and to maintain adequate protection against risks. We also outline the potential benefit of this research and describe the importance of the knowledge to be gained. All investigators involved in this application have completed or will complete training on the protection of human subjects before the project begins.

Potential Benefits of the Proposed Research to Research Participants and Others

Patients may benefit from improved adherence or increased communication with clinic staff, easing their visit adherence or transfer reporting. Information obtained in this study will be used by the MOHand its partners to inform retention efforts, which may lead to improving ART retention and thus HIV prevention.

Importance of the Knowledge to be Gained

The risks are offset by the potential value of the information to be gained in this study. This project will evaluate the effectiveness, feasibility, and acceptability of a promising intervention to improve ART retention at reduced cost and workload. If successful, both providers and patients would encounter distinct advantages. We will integrate 2wT into EMRS to improve and ease reporting. These advantages could be scaled up if the approach is adopted by the MoH, providing population-levels gains to thousands of LH/MPC patients and, ultimately, millions of patients in regional ART programs. If texting with ART clients to increase retention is found to be better than routine to prevent default and improve data, scale up could dramatically reduce time spent on unnecessary tracing and decrease the burden on B2C staff. Future funding could enable scale-up of the intervention in other high volume ART sites with an EMRS, conducting additional rigorous evaluation in diverse field and clinical contexts. We would seek funding to test 2wT among rural populations or smaller urban areas – perhaps testing the advantages of only 2WT without the EMRS linkage for sites without EMRS options.

Dissemination

This project will estimate the effectiveness, feasibility, and acceptability of a two-way texting (2WT) follow-up to improve ART retention. We will conduct an RCT and then expand in routine care in two large public ART clinics in Lilongwe Malawi.

If successful, patients would be retained in care at lower cost and reduced healthcare worker workload, providing distinct advantages to both groups. These advantages could be scaled up if the approach is routinized with help of 2WT to electronic medical records system (EMRS) integration, providing population-levels gains to millions of providers and patients. Publications resulting from this study will adhere to human subjects rules and regulations for this low-risk, non-research determination are adhered to.

Ghant chart of RCT research activities

	Completed	RCT			
		Q1	Q2	Q3	Q4
Previous 2wT study					
UW & Malawi: Review and approve protocol and materials	X				
Formative research, user-feedback, message testing	X				
Revise SOPs, data collection instruments, database	X				
Train local teams in 2wT	X				
Implement 2wT among new MPC ART clients	X				
**Pragmatic RCT at Lighthouse and MPC** with **routine care teams**					
Review and approve protocol and materials		X			
Train study team in RCT protcols		X			
Randomize 2wT enrollment among those eligible		X	X	X	
Those randomized to routine start 2wT at 6 months				X	X
Stakeholder engagement and process eval updates					X
Analysis of main RCT outcomes					X
Publication and dissemination					x

Appendices:

Appendix 1: Client consent for routine 2wT, year 2

INFORMED CONSENT FOR PARTICIPANTS

Two-way texting for Loss to Follow-up Prevention Study

Version 1.0, Dated 04-15-2020

**PRINCIPAL INVESTIGATOR**	**Phone and Address**
Dr. Hannock Tweya	0999 386 715, Lighthouse Trust, Box 106, Lilongwe

INTRODUCTION:

You are being asked to take part in the research study named above. This study is being conducted at Lighthouse Trust clinics in Lilongwe. The persons in charge of the study is Dr. Hannock Tweya.

You should only participate if you want to; choosing not to take part will not disadvantage you in any way. Before you decide whether you want to take part, it is important for you to understand, why the research is being done and what your participation will involve. Please feel free to ask us if there is anything unclear or if you would like more information. Once you understand the study, and if you agree to take part, you will be asked to sign this consent form or make you mark in front of someone. You will be offered a copy to keep.

Please note that:

- Your participation in this research is entirely voluntary;
- You may decide not to participate or to withdraw from the study at any time without losing the benefits of being treated at this clinic.
- If you decide not to participate in this study, you can still join another research study later, if one is available and you qualify.

PURPOSE OF THE STUDY:

Loss to follow up from HIV care is a problem in this clinic and in Malawi. The purpose of this study is to find out if two-way texting – where we can text you and you can text us back – could help us better keep our patients in care. Moreover, we want to find out if sending patient reminders of their visits could encourage patients to return to the clinic, and, if patients transfer, they could tell us by text. We also want to see if we could change appointment date by text, making it easier for patients who cannot make their appointments. Approximately 500 participants will take part in this study.

STUDY PROCEDURES:

You will be given all information you need to decide if you want to participate in this study. If you decide to participate in this study, we will register your phone number into the texting system. We will test your phone to make sure you get the message. Then, if you start HIV medication, you will receive a weekly message on your phone with an encouraging message, like: "This is your reminder." "Thank you for staying healthy!" or "You're making great choices every day!". You will also receive a reminder of your visit 3 and 1 day before the visit, and you will tell us if you can come for your visit or not. The message will say, "You have a visit in 3 days. See you soon! If you will return, enter 1=Yes or 0=No and press send." If you tell us you will not return, we will ask you if you need to change your appointment or if you started receiving ARVs from another clinic. You will be texting with a member of the Lighthouse team or we can call you back if you prefer. The messages will not contain information about your HIV status or ARVs. Sending us messages to this number is free. We will not send messages if you do not start HIV medication.

We will send these weekly messages and visit reminders by text for 12 months. You may decide not to take part in the study at any time.

We will ask about 100 participants to tell us how they feel about the texting system at ART visits at 3 months or 6-months. We would like to know your opinions on the texts and using the texting for visit confirmation. This may help us improve the system.

The research team will use the routine clinic data and your responses to these messages as part of the study. The information in the texting system uses your Lighthouse patient ID. Data will be saved on central computers and in an online database that will be analysed by authorized individuals (e.g. researchers) other than the usual healthcare provider. Your privacy will be protected and other people cannot identify you.

POTENTIAL RISKS /BENEFITS:

There is a risk that others who see your phone may find out your HIV status and/or ART use as a result of these texts. That could cause problems between you and your sexual partners, including the potential for physical, verbal, sexual, or economic harm. Aside from the reminders, there are no other direct benefits to you by participating in this study. We believe that the healthcare providers can profit from the knowledge we gain from this study. People who see your phone may see that you have an appointment, but no texts will contain any HIV information that could disclose your status.

CONFIDENTIALITY:

Your identity will be kept confidential by using your Lighthouse ID number, which will be used to identify you, and we will only be using that number through the record keeping. You have the right to have insight in your data if you wish so. The information leaving the health clinic for research purposes will be contained within an online database that can be viewed by study researchers in the United States. This database is encrypted and password protected. Only coded study data

will be used for analyses in the United States. If you withdraw from the study, the data that is collected before the withdraw your consent will be used in the study.

We have a Certificate of Confidentiality from the United States from the National Institutes of Health. These protections only apply to data held in the United States. This helps us protect your privacy. The certificate means that we do not have to give out information, documents, or samples that could identify you even if we are asked to by a court of law in the United States. We will use the Certificate to resist any demands for identifying information.

We can't use the Certificate to withhold your research information if you give your written consent to give it to an insurer, employer, or other person. Also, you or a member of your family can share information about yourself or your part in this research if you wish.

There are some limits to this protection. We will voluntarily provide the information to:

- a member of the United States government who needs it in order to audit or evaluate the research;
- individuals at the institution(s) conducting the research, the funding agency, and other groups involved in the research, if they need the information to make sure the research is being done correctly;
- individuals who want to conduct secondary research if allowed by federal regulations and according to your consent for future research use as described in this form;
- to relevant authorities as required by other Federal, State, or local laws.

The Certificate expires when the NIH funding for this study ends. Currently this is June 30, 2022. Any data collected after expiration is not protected as described above. Data collected prior to expiration will continue to be protected

ALTERNATIVES TO PARTICIPATION:

It is up to you to decide whether or not to take part in this study. If you do decide to take part you will be given this form to keep and be asked to sign a consent form once you are enrolled. If you do not wish to participate, you may have routine care, including routine Back to Care tracing services.

FUTURE USE

The information that we obtain from you for this study might be used for future studies. We may remove anything that might identify you from the information and specimens. If we do so, that information may then be used for future research studies or given to another investigator without getting additional permission from you. It is also possible that in the future we may want to use or share study information that might identify you. If we do, a review board will decide whether or not we need to get additional permission from you.

PERSONS TO CONTACT FOR PROBLEMS OR QUESTIONS:

All research on human volunteers is reviewed by the Malawi Health Sciences Research Committee, a committee that works to protect your rights and welfare. If you have questions about the research or your rights as a research participant, you can contact:

The Chairman
National Health Science and Research Committee
P.O Box 30377
Lilongwe
Tel +265789400
Cell+265994063425

Dr Hannock Tweya
Lighthouse Trust
Kamuzu Central Hospital
P. O Box 106
Tel +2651758940
htweya@lighthouse.org.mw

SIGNATURE PAGE

INFORMED CONSENT FOR PARTICIPANTS
Two-way texting for Loss to Follow-up Prevention Study
Version 1.0, Dated 04-15-2020

STATEMENT OF CONSENT

Thank you for considering taking part in this research. The person organizing the research must explain the project to you before you agree to take part. If you have any questions arising from the informed consent sheet or explanation already given to you, please ask the researcher before you decide whether to join in. You will be given a copy of this consent form to keep and refer to at any time.

Please tick or initial

1. I have read the informed consent or had it read and explained to me. I understand the information and I voluntarily agree to join this study. ☐
2. I consent that medical data that has been collected by my antiretroviral therapy providers can be further used in this study. ☐
3. I consent that my medical records may be examined and statistically analysed by authorized individuals other than my usual healthcare provider. ☐
4. I consent that data collected during the study will be stored on central computers and online to be used for review, while kept strictly confidential. ☐
5. I understand that if I decide at any time during the research that I no longer wish to participate in this project, I can notify the researchers involved and withdraw from it immediately without giving any reason. ☐

Participant's Statement:

____
Name

______________________ __________________ ☐
Signature Date Thumbprint

Investigator's Statement: I ____________confirm that I carefully explained the nature, demands and any foreseeable risks (where applicable) of the proposed research to the participant.

_________________________ ____________________

Signature Date

Witness to consent if Subject Unable to Read or Write (Must be different from the person obtaining consent):

____

Name

_________________________ ____________________

Signature Date

Appendix 1a: Client consent for RCT

INFORMED CONSENT FOR PARTICIPANTS

Two-way texting for Loss to Follow-up Prevention Study

Version 1.0, Dated 09-03-2022

PRINCIPAL INVESTIGATOR	Phone and Address
Dr. Hannock Tweya	0999 386 715, Lighthouse Trust, Box 106, Lilongwe

INTRODUCTION:

You are being asked to take part in the research study named above. This study is being conducted at Lighthouse Trust clinics in Lilongwe. The persons in charge of the study is Dr. Hannock Tweya.

You should only participate if you want to; choosing not to take part will not disadvantage you in any way. Before you decide whether you want to take part, it is important for you to understand, why the research is being done and what your participation will involve. Please feel free to ask us if there is anything unclear or if you would like more information. Once you understand the study, and if you agree to take part, you will be asked to sign this consent form or make you mark in front of someone. You will be offered a copy to keep.

Please note that:

- Your participation in this research is entirely voluntary;
- You may decide not to participate or to withdraw from the study at any time without losing the benefits of being treated at this clinic.
- If you decide not to participate in this study, you can still join another research study later, if one is available and you qualify.

PURPOSE OF THE STUDY:

Loss to follow up from HIV care is a problem in this clinic and in Malawi. The purpose of this study is to find out if two-way texting – where we can text you and you can text us back – could help us better keep our patients in care. Moreover, we want to find out if sending patient reminders of their visits could encourage patients to return to the clinic, and, if patients transfer, they could tell us by text. We also want to see if we could change appointment date by text, making it easier for patients who cannot make their appointments. Approximately 410 participants will take part in this

study. About half will be in texting now. The other half will start with texting reminders in 6 months.
.

STUDY PROCEDURES:

You will be given all information you need to decide if you want to participate in this study. Participants will be enrolled in either a texting group or to routine care for 6 months. You will not get to choose which group you are in. It will be assigned to you. At 6 months, everyone will receive reminder texts. If you decide to participate in this study, we will register your phone number into the texting system. In both groups, we will test your phone now to make sure you get the message.

Then, if you start HIV medication, and you are in the routine group, you will start receiving these messages in 6 months. If you start HIV medication, and you are in the texting group, you will receive a weekly message on your phone with an encouraging message, like: “This is your reminder.” “Thank you for staying healthy!” or “You’re making great choices every day!”. You will also receive a reminder of your visit 3 and 1 day before the visit, and you will tell us if you can come for your visit or not. The message will say, “You have a visit in 3 days. See you soon! If you will return, enter 1=Yes or 0=No and press send.” If you tell us you will not return, we will ask you if you need to change your appointment or if you started receiving ARVs from another clinic. You will be texting with a member of the Lighthouse team or we can call you back if you prefer. The messages will not contain information about your HIV status or ARVs. Sending us messages to this number is free. We will not send messages if you do not start HIV medication.

We will send these weekly messages and visit reminders by text for 12 months. You may decide not to take part in the study at any time.

The research team will use the routine clinic data and your responses to these messages as part of the study. The information in the texting system uses your Lighthouse patient ID. Data will be saved on central computers and in an online database that will be analysed by authorized individuals (e.g. researchers) other than the usual healthcare provider. Your privacy will be protected and other people cannot identify you.

POTENTIAL RISKS /BENEFITS:

There is a risk that others who see your phone may find out your HIV status and/or ART use as a result of these texts. That could cause problems between you and your sexual partners, including the potential for physical, verbal, sexual, or economic harm. Aside from the reminders, there are no other direct benefits to you by participating in this study. We believe that the healthcare providers can profit from the knowledge we gain from this study. People who see your phone may see that you have an appointment, but no texts will contain any HIV information that could disclose your status.

CONFIDENTIALITY:

Your identity will be kept confidential by using your Lighthouse ID number, which will be used to identify you, and we will only be using that number through the record keeping. You have the right to have insight in your data if you wish so. The information leaving the health clinic for research purposes will be contained within an online database that can be viewed by study researchers in the United States. This database is encrypted and password protected. Only coded study data will be used for analyses in the United States. If you withdraw from the study, the data that is collected before the withdraw your consent will be used in the study.

We have a Certificate of Confidentiality from the United States from the National Institutes of Health. These protections only apply to data held in the United States. This helps us protect your privacy. The certificate means that we do not have to give out information, documents, or samples that could identify you even if we are asked to by a court of law in the United States. We will use the Certificate to resist any demands for identifying information.

We can't use the Certificate to withhold your research information if you give your written consent to give it to an insurer, employer, or other person. Also, you or a member of your family can share information about yourself or your part in this research if you wish.

There are some limits to this protection. We will voluntarily provide the information to:

- a member of the United States government who needs it in order to audit or evaluate the research;
- individuals at the institution(s) conducting the research, the funding agency, and other groups involved in the research, if they need the information to make sure the research is being done correctly;
- individuals who want to conduct secondary research if allowed by federal regulations and according to your consent for future research use as described in this form;
- to relevant authorities as required by other Federal, State, or local laws.

The Certificate expires when the NIH funding for this study ends. Currently this is June 30, 2023. Any data collected after expiration is not protected as described above. Data collected prior to expiration will continue to be protected

ALTERNATIVES TO PARTICIPATION:

It is up to you to decide whether or not to take part in this study. If you do decide to take part you will be given this form to keep and be asked to sign a consent form once you are enrolled. If you do not wish to participate, you may have routine care, including routine Back to Care tracing services.

FUTURE USE

The information that we obtain from you for this study might be used for future studies. We may remove anything that might identify you from the information and specimens. If we do so, that information may then be used for future research studies or given to another investigator without

getting additional permission from you. It is also possible that in the future we may want to use or share study information that might identify you. If we do, a review board will decide whether or not we need to get additional permission from you.

PERSONS TO CONTACT FOR PROBLEMS OR QUESTIONS:

All research on human volunteers is reviewed by the Malawi Health Sciences Research Committee, a committee that works to protect your rights and welfare. If you have questions about the research or your rights as a research participant, you can contact:

The Chairman
National Health Science and Research Committee
P.O Box 30377
Lilongwe
Tel +265789400
Cell+265994063425

Dr Jacqueline Huwa
Lighthouse Trust
Kamuzu Central Hospital
P. O Box 106
Tel +2651758940
jhuwa@lighthouse.org.mw

SIGNATURE PAGE

INFORMED CONSENT FOR PARTICIPANTS
Two-way texting for Loss to Follow-up Prevention Study
Version 1.0, Dated 04-15-2020

STATEMENT OF CONSENT

Thank you for considering taking part in this research. The person organizing the research must explain the project to you before you agree to take part. If you have any questions arising from the informed consent sheet or explanation already given to you, please ask the researcher before you decide whether to join in. You will be given a copy of this consent form to keep and refer to at any time.

Please tick or initial

6. I have read the informed consent or had it read and explained to me. I understand the information and I voluntarily agree to join this study. ☐
7. I consent that medical data that has been collected by my antiretroviral therapy providers can be further used in this study. ☐
8. I consent that my medical records may be examined and statistically analysed by authorized individuals other than my usual healthcare provider. ☐
9. I consent that data collected during the study will be stored on central computers and online to be used for review, while kept strictly confidential. ☐
10. I understand that if I decide at any time during the research that I no longer wish to participate in this project, I can notify the researchers involved and withdraw from it immediately without giving any reason. ☐

Participant’s Statement: ☐

____
Name

_____________________________ ________________________
Signature Date Thumbprint

Investigator's Statement: I ____________confirm that I carefully explained the nature, demands and any foreseeable risks (where applicable) of the proposed research to the participant.

_____________________________ ________________________
Signature Date

Witness to consent if Subject Unable to Read or Write (Must be different from the person obtaining consent):

____
Name

_____________________________ ________________________
Signature Date

References

1. UNAIDS. *Country Overview: Malawi*. 2018 [cited 2019 November 6]; Available from: https://www.unaids.org/en/regionscountries/countries/malawi.
2. Fox, M.P. and S. Rosen, *Retention of Adult Patients on Antiretroviral Therapy in Low- and Middle-Income Countries: Systematic Review and Meta-analysis 2008-2013.* Journal of acquired immune deficiency syndromes (1999), 2015. **69**(1): p. 98-108.
3. Cohen, M.S., et al., *Prevention of HIV-1 infection with early antiretroviral therapy.* New England journal of medicine, 2011. **365**(6): p. 493-505.
4. Tanser, F., et al., *High coverage of ART associated with decline in risk of HIV acquisition in rural KwaZulu-Natal, South Africa.* Science, 2013. **339**(6122): p. 966-971.
5. Douglas, G.P., et al., *Using touchscreen electronic medical record systems to support and monitor national scale-up of antiretroviral therapy in Malawi.* PLoS medicine, 2010. **7**(8): p. e1000319.
6. Douglas, G.P., Z. Landis-Lewis, and H. Hochheiser, *Simplicity and usability: lessons from a touchscreen electronic medical record system in Malawi.* interactions, 2011. **18**(6): p. 50-53.
7. Landis Lewis, Z., et al., *The feasibility of automating audit and feedback for ART guideline adherence in Malawi.* Journal of the American Medical Informatics Association, 2011. **18**(6): p. 868-874.
8. McKay, M.V. and G.P. Douglas. *Touchscreen clinical workstations at point of care: A paradigm shift in electronic medical record design for developing countries*. in *2008 5th IET Seminar on Appropriate Healthcare Technologies for Developing Countries*. 2008. IET.
9. Waters, E., et al. *Experience implementing a point-of-care electronic medical record system for primary care in Malawi*. in *Medinfo*. 2010.
10. Joint United Nations Programme on HIV/AIDS, *On the fast-track to an AIDS-free generation. Geneva: UNAIDS; 2016*. 2019.
11. Tenthani, L., et al., *Retention in care under universal antiretroviral therapy for HIV infected pregnant and breastfeeding women ("Option B+") in Malawi.* AIDS (London, England), 2014. **28**(4): p. 589.
12. Weigel, R., et al., *Outcomes and associated risk factors of patients traced after being lost to follow-up from antiretroviral treatment in Lilongwe, Malawi.* BMC infectious diseases, 2011. **11**(1): p. 31.
13. Tabatabai, J., et al., *Understanding reasons for treatment interruption amongst patients on antiretroviral therapy–a qualitative study at the Lighthouse Clinic, Lilongwe, Malawi.* Global health action, 2014. **7**(1): p. 24795.
14. Haddad, L.B., et al., *Factors associated with condom use among men and women living with HIV in Lilongwe, Malawi: a cross-sectional study.* BMJ sexual & reproductive health, 2018. **44**(1): p. 1-12.
15. Feldacker, C., et al., *Who starts? Factors associated with starting antiretroviral therapy among eligible patients in two, public HIV clinics in Lilongwe, Malawi.* PloS one, 2012. **7**(11): p. e50871.
16. Ardura-Garcia, C., et al., *Implementation and operational research: early tracing of children lost to follow-up from antiretroviral treatment: true outcomes and future risks.* Journal of acquired immune deficiency syndromes (1999), 2015. **70**(5): p. e160.
17. Estill, J., et al., *Tracing of patients lost to follow-up and HIV transmission: mathematical modelling study based on two large ART programmes in Malawi.* Journal of acquired immune deficiency syndromes (1999), 2014. **65**(5): p. e179.

18. Tweya, H., et al., *Early active follow-up of patients on antiretroviral therapy (ART) who are lost to follow-up: the 'Back-to-Care' project in Lilongwe, Malawi.* Tropical Medicine & International Health, 2010. **15**: p. 82-89.
19. Chammartin, F., et al., *Outcomes of patients lost to follow-up in African antiretroviral therapy programs: individual patient data meta-analysis.* Clinical infectious diseases, 2018. **67**(11): p. 1643-1652.
20. Tweya, H., et al., *Loss to follow-up before and after initiation of antiretroviral therapy in HIV facilities in Lilongwe, Malawi.* PloS one, 2018. **13**(1): p. e0188488.
21. Rosen, S. and M. Ketlhapile, *Cost of using a patient tracer to reduce loss to follow-up and ascertain patient status in a large antiretroviral therapy program in Johannesburg, South Africa.* Tropical Medicine & International Health, 2010. **15**: p. 98-104.
22. Georgette, N., et al., *Impact of a clinical program using weekly Short Message Service (SMS) on antiretroviral therapy adherence support in South Africa: a retrospective cohort study.* BMC medical informatics and decision making, 2017. **17**(1): p. 18.
23. Georgette, N., et al., *The acceptability and perceived usefulness of a weekly clinical SMS program to promote HIV antiretroviral medication adherence in KwaZulu-Natal, South Africa.* AIDS and Behavior, 2016. **20**(11): p. 2629-2638.
24. Kemp, C.G. and J. Velloza, *Implementation of eHealth interventions across the HIV care cascade: a review of recent research.* Current HIV/AIDS Reports, 2018. **15**(6): p. 403-413.
25. Govindasamy, D., et al., *Interventions to improve or facilitate linkage to or retention in pre-ART (HIV) care and initiation of ART in low-and middle-income settings–a systematic review.* Journal of the International AIDS Society, 2014. **17**(1): p. 19032.
26. International Telecommunication Union. *Mobile cellular subscriptions (per 100 people)*. International Telecommunication Union, World Telecommunication/ICT Development Report and database 2017 [cited 2019 January 3]; Available from: https://data.worldbank.org/indicator/IT.CEL.SETS.P2?locations=ZW.
27. Watkins, J.O.T.A., et al., *Mobile phone use among patients and health workers to enhance primary healthcare: A qualitative study in rural South Africa.* Social Science & Medicine, 2018. **198**: p. 139-147.
28. Organization, W.H., *Consolidated guidelines on general HIV care and the use of antiretroviral drugs for treating and preventing HIV infection: recommendations for a public health approach.* Geneva: World Health Organization, 2013: p. 269.
29. Fairbanks, J., et al., *You will know that despite being HIV positive you are not alone: qualitative study to inform content of a text messaging intervention to improve prevention of mother-to-child HIV transmission.* JMIR mHealth and uHealth, 2018. **6**(7): p. e10671.
30. Crankshaw, T., et al., *Exploring the patterns of use and the feasibility of using cellular phones for clinic appointment reminders and adherence messages in an antiretroviral treatment clinic, Durban, South Africa.* AIDS patient care and STDs, 2010. **24**(11): p. 729-734.
31. Murenje, V., et al., *Feasibility and acceptability of a two-way texting intervention for post-operative follow up for voluntary medical male circumcision*, in *20th ICASA International Conference on AIDS and STI's in Africa*. 2019: Kigali, Rwanda.
32. Nachega, J.B., et al., *Acceptability and feasibility of mHealth and community-based directly observed antiretroviral therapy to prevent mother-to-child HIV transmission in South African pregnant women under Option B+: an exploratory study.* Patient preference and adherence, 2016. **10**: p. 683.

33. Linnemayr, S., et al., *Text messaging for improving antiretroviral therapy adherence: no effects after 1 year in a randomized controlled trial among adolescents and young adults.* American journal of public health, 2017. **107**(12): p. 1944-1950.
34. Mbuagbaw, L., et al., *The Cameroon Mobile Phone SMS (CAMPS) trial: a randomized trial of text messaging versus usual care for adherence to antiretroviral therapy.* PloS one, 2012. **7**(12): p. e46909.
35. Kliner, M., et al., *Using no-cost mobile phone reminders to improve attendance for HIV test results: a pilot study in rural Swaziland.* Infectious diseases of poverty, 2013. **2**(1): p. 12-12.
36. Davey, D.L.J., et al., *Why wait? We need to scale-up infant male circumcision for global HIV control.* AIDS, 2016. **30**(11): p. 1847-1848.
37. Mayer, J.E. and P. Fontelo, *Meta-analysis on the effect of text message reminders for HIV-related compliance.* AIDS Care, 2017. **29**(4): p. 409-417.
38. Ronen, K., et al., *SMS messaging to improve ART adherence: perspectives of pregnant HIV-infected women in Kenya on HIV-related message content.* AIDS care, 2018. **30**(4): p. 500-505.
39. Jong, S., Y. Cuca, and L.M. Thompson, *Meta-analysis of mobile phone reminders on HIV patients' retention to care.* Journal of mobile technology in medicine, 2017. **6**(1): p. 5.
40. Amico, K.R., *Evidence for technology interventions to promote ART adherence in adult populations: a review of the literature 2012–2015.* Current HIV/AIDS Reports, 2015. **12**(4): p. 441-450.
41. Kanters, S., et al., *Interventions to improve adherence to antiretroviral therapy: a systematic review and network meta-analysis.* The lancet HIV, 2017. **4**(1): p. e31-e40.
42. Finitsis, D.J., J.A. Pellowski, and B.T. Johnson, *Text message intervention designs to promote adherence to antiretroviral therapy (ART): a meta-analysis of randomized controlled trials.* PloS one, 2014. **9**(2): p. e88166.
43. Amankwaa, I., et al., *Effectiveness of short message services and voice call interventions for antiretroviral therapy adherence and other outcomes: A systematic review and meta-analysis.* PloS one, 2018. **13**(9): p. e0204091.
44. Lester, R.T., et al., *Effects of a mobile phone short message service on antiretroviral treatment adherence in Kenya (WelTel Kenya1): a randomised trial.* The Lancet, 2010. **376**(9755): p. 1838-1845.
45. Horvath, T., et al., *Mobile phone text messaging for promoting adherence to antiretroviral therapy in patients with HIV infection.* Cochrane Database of Systematic Reviews, 2012(3).
46. L'Engle, K.L., et al., *Evaluating feasibility, reach and potential impact of a text message family planning information service in Tanzania.* Contraception, 2013. **87**(2): p. 251-256.
47. Ware, N.C., et al., *The Meanings in the messages: how SMS reminders and real-time adherence monitoring improve antiretroviral therapy adherence in rural Uganda.* AIDS (London, England), 2016. **30**(8): p. 1287.
48. Smith, J., A. Braunack-Mayer, and G. Wittert, *What do we know about men's help-seeking and health service use?* 2006.
49. Sharma, M., et al., *Modeling the cost-effectiveness of home-based HIV testing and education (HOPE) for pregnant women and their male partners in Nyanza Province, Kenya.* Journal of acquired immune deficiency syndromes (1999), 2016. **72**(Suppl 2): p. S174.
50. Shand, T., et al., *The HIV blind spot: men and HIV testing, treatment and care in sub-Saharan Africa.* IDS Bulletin, 2014. **45**(1): p. 53-60.

51. McLaren, Z.M., C. Ardington, and M. Leibbrandt, *Distance decay and persistent health care disparities in South Africa.* BMC health services research, 2014. **14**(1): p. 541.
52. Pop-Eleches, C., et al., *Mobile phone technologies improve adherence to antiretroviral treatment in a resource-limited setting: a randomized controlled trial of text message reminders.* AIDS (London, England), 2011. **25**(6): p. 825.
53. DeRenzi, B., et al. *Improving community health worker performance through automated SMS*. in *Proceedings of the fifth international conference on information and communication technologies and development*. 2012. ACM.
54. Perrier, T., et al. *Engaging pregnant women in Kenya with a hybrid computer-human SMS communication system*. in *Proceedings of the 33rd Annual ACM Conference on Human Factors in Computing Systems*. 2015. ACM.
55. Wald, D.S., S. Butt, and J.P. Bestwick, *One-way versus two-way text messaging on improving medication adherence: meta-analysis of randomized trials.* The American journal of medicine, 2015. **128**(10): p. 1139. e1-1139. e5.
56. Van der Kop, M.L., et al., *In-depth analysis of patient-clinician cell phone communication during the WelTel Kenya1 antiretroviral adherence trial.* PLoS One, 2012. **7**(9): p. e46033.
57. Gimbel, S., et al., *A Missing Link: HIV-/AIDS-Related mHealth Interventions for Health Workers in Low-and Middle-Income Countries.* Current HIV/AIDS Reports, 2018. **15**(6): p. 414-422.
58. Feldacker, C., et al., *Reducing provider workload while preserving patient safety via a two-way texting intervention in Zimbabwe's voluntary medical male circumcision program: study protocol for an un-blinded, prospective, non-inferiority, randomized controlled trial.* Trials, 2019. **20**(1): p. 451.
59. Feldacker, C., et al., *Reducing Provider Workload While Preserving Patient Safety: A Randomized Control Trial Using 2-Way Texting for Postoperative Follow-up in Zimbabwe's Voluntary Medical Male Circumcision Program.* JAIDS Journal of Acquired Immune Deficiency Syndromes, 2020. **83**(1): p. 16-23.
60. Feldacker, C., et al., *Usability and acceptability of a two-way texting intervention for post-operative follow-up for voluntary medical male circumcision in Zimbabwe.* PLOS ONE, 2020. **15**(6): p. e0233234.
61. Aranda-Jan, C.B., N. Mohutsiwa-Dibe, and S. Loukanova, *Systematic review on what works, what does not work and why of implementation of mobile health (mHealth) projects in Africa.* BMC public health, 2014. **14**(1): p. 188.
62. DeRenzi, B., et al., *Mobile Phone Tools for Field-Based Health care Workers in Low-Income Countries.* Mount Sinai Journal of Medicine: A Journal of Translational and Personalized Medicine, 2011. **78**(3): p. 406-418.
63. Thirumurthy, H. and R.T. Lester, *M-health for health behaviour change in resource-limited settings: applications to HIV care and beyond.* Bulletin of the World Health Organization, 2012. **90**(5): p. 390-392.
64. Banks, K., S.M. McDonald, and F. Scialom, *Mobile technology and the last mile:"Reluctant innovation" and FrontlineSMS.* innovations, 2011. **6**(1): p. 7-12.
65. Medic Mobile. *Medic Mobile For Antenatal Care: Do-It-Yourself Toolkit*. 2016 [cited 2016 August 22]; Available from: http://medicmobile.org/diy.
66. West, D., *How mobile devices are transforming healthcare.* Issues in technology innovation, 2012. **18**(1): p. 1-11.
67. Pathak, P., *India Vaccination Pilot Progress Report.* Medic Mobile and Clif Bar, 2012.

68. Medic Mobile. *Drug Stock Monitoring*. 2016 [cited 2016 August 22]; Available from: http://medicmobile.org/usecases/drug-stock-monitoring.
69. Jumreornvong, O., *New Weapon In The War Against AIDS: Your Mobile Phone.* Intersect: The Stanford Journal of Science, Technology and Society, 2014. **7**(1).
70. World Health Organization *Classification of digital health interventions v1. 0: a shared language to describe the uses of digital technology for health*. 2018.
71. Director, N.I.O.H.O.O.T., *Fast Healthcare Interoperability Resources (FHIR®) Standard*, in *Notice Number: NOT-OD-19-122*. 2019, National Institutes of Health: Bethesda, MD.
72. Kunutsor, S., et al., *Using mobile phones to improve clinic attendance amongst an antiretroviral treatment cohort in rural Uganda: a cross-sectional and prospective study.* AIDS and behavior, 2010. **14**(6): p. 1347-1352.
73. Siedner, M.J., J.E. Haberer, and D.R. Bangsberg, *Know your audience: predictors of success for a patient-centered texting app to augment linkage to HIV care in rural Uganda.* Journal of medical Internet research, 2015. **17**(3): p. e78.
74. Organization, W.H., *Consolidated guidelines on the use of antiretroviral drugs for treating and preventing HIV infection: recommendations for a public health approach*. 2016: World Health Organization.
75. President's Emergency Plan for AIDS Relief, *PEPFAR Monitoring, Evaluation, and Reporting Indicator Reference Guide.* 2015, PEPFAR: Washington DC.
76. Losina, E., et al., *Cost-effectiveness of preventing loss to follow-up in HIV treatment programs: a Cote d'Ivoire appraisal.* PLoS medicine, 2009. **6**(10): p. e1000173.
