# Supplementary material for: Evaluating the effect of interactive two-way texting on 6-month antiretroviral therapy outcomes: Findings from a randomized controlled trial in Lilongwe, Malawi
Source: PLOS Glob Public Health. 2025 Sep 10;5(9):e0004598. doi: 10.1371/journal.pgph.0004598 (PMC12422422; doi:10.1371/journal.pgph.0004598)
Supplement: S1 Table — (DOCX) [file pgph.0004598.s002.docx]

## ***S1 Table: CONSORT 2022-Outcomes Checklist***

| **Section** | **Item No.** | **CONSORT 2010 Item** | **CONSORT-Outcomes 2022 item** | **Location Reported^b^** |
| --- | --- | --- | --- | --- |
| **Title and abstract** | | | | |
|  | 1a | Identification as a randomized trial in the title | - | 1 |
|  | 1b | Structured summary of trial design, methods, results, and conclusions (for specific guidance see CONSORT for  abstracts) | - | 2 |
| **Introduction** | | | | |
| Background and objectives | 2a | Scientific background and explanation of rationale | - | 3-4 |
|  | 2b | Specific objectives or hypotheses | - | 4 |
| **Methods** | | | | |
| Trial design | 3a | Description of trial design (such as parallel, factorial) including  allocation ratio | - | 4-5 |
| Participants | 4a | Eligibility criteria for participants | - | 5 |
|  | 4b | Settings and locations where the data were collected | - | 4 |
| Interventions | 5 | The interventions for each group with sufficient details to allow replication, including how and when they were actually administered (for specific guidance see TIDieR checklist  and guide) | - | 5-6 |
| Outcomes | 6a | Completely defined prespecified primary and secondary outcome measures,  including how and when they were assessed | - | 7 |

| Sample size | 7a | How sample size was determined | - | 5 |
| --- | --- | --- | --- | --- |

|  | 7a.1 |  | Define and justify the target difference between treatment groups (eg, the minimal  important difference) | 5-6 |
| --- | --- | --- | --- | --- |
| **Randomization** | | | | |
| Sequence generation | 8a | Method used to generate the random allocation sequence | - | 5 |
|  | 8b | Type of randomization; details of any restriction (such as blocking and block size) | - | 5 |
| Allocation concealment mechanism | 9 | Mechanism used to implement the random allocation sequence (such as sequentially numbered containers), describing any steps taken to conceal the sequence until  interventions were assigned | - | 5 |
| Statistical methods | 12a | Statistical methods used to compare groups for primary and secondary outcomes | - | 7 |
|  | 12a.2 |  | State and justify any criteria for excluding any outcome data from the analysis and reporting, or report that no outcome data  were excluded | 7 |

|  | 12a.4 |  | Provide a definition of the outcome analysis population relating to nonadherence of the trial protocol (eg, as a  randomized analysis) | 4-6 |
| --- | --- | --- | --- | --- |
|  | 12b | Methods for additional analyses, such as subgroup analyses and adjusted  analyses | - | 7 |
| **Results** | | | | |
| Participant flow (a diagram is strongly recommended) | 13a | For each group, the numbers of participants who were randomly assigned, received intended treatment, and were analyzed for the primary outcome | - | 8, S1 |
|  | 13b | For each group, losses and exclusions after randomization,  together with reasons | - | 8, S1 |
| Recruitment | 14a | Dates defining the periods of recruitment and follow-up | - | 5 |
|  | 14b | Why the trial ended or was stopped | - | 5 |
| Baseline data | 15 | A table showing baseline demographic and clinical characteristics for each group | - | 8 |
| Numbers analyzed | 16 | For each group, number of participants (denominator) included in each analysis and whether the analysis was by  original assigned groups | - | 8 |
| Outcomes and estimation | 17a | For each primary and secondary outcome, results for each group, and the estimated effect size and its precision  (such as 95% CI) | - | 9-11 |
|  | 17b | For binary outcomes, presentation of both absolute and relative effect sizes is  recommended | - | 9 |
| Ancillary analyses | 18 | Results of any other analyses performed, including subgroup  analyses and adjusted analyses, distinguishing prespecified from exploratory | - | 9-11, S3 |

| **Discussion** | | | | |
| --- | --- | --- | --- | --- |
| Limitations | 20 | Trial limitations, addressing sources of potential bias, imprecision, and, if relevant,  multiplicity of analyses | - | 12 |
| Generalizability | 21 | Generalizability (external validity, applicability) of the trial  findings | - | 12 |
| Interpretation | 22 | Interpretation consistent with results, balancing benefits and harms, and considering other relevant evidence | - | 11-13 |
| **Other Information** | | | | |
| Registration | 23 | Registration number and name of trial registry | - | 5 |
| Funding | 25 | Sources of funding and other  support (such as supply of drugs), role of funders | - | 14 |
