# Supplementary material for: Evaluating the effect of interactive two-way texting on 6-month antiretroviral therapy outcomes: Findings from a randomized controlled trial in Lilongwe, Malawi
Source: PLOS Glob Public Health. 2025 Sep 10;5(9):e0004598. doi: 10.1371/journal.pgph.0004598 (PMC12422422; doi:10.1371/journal.pgph.0004598)

## ***S1 Fig: CONSORT Diagram Study Participant Flow***

Out of 1,091 new ART clients screened at MPC and LH, 467 (43%) met the eligibility criteria and were included (S1 Fig). Lack of phone access (313, 50%), illiteracy (173, 28%), and being under 18 years (40, 6%) were barriers to inclusion.


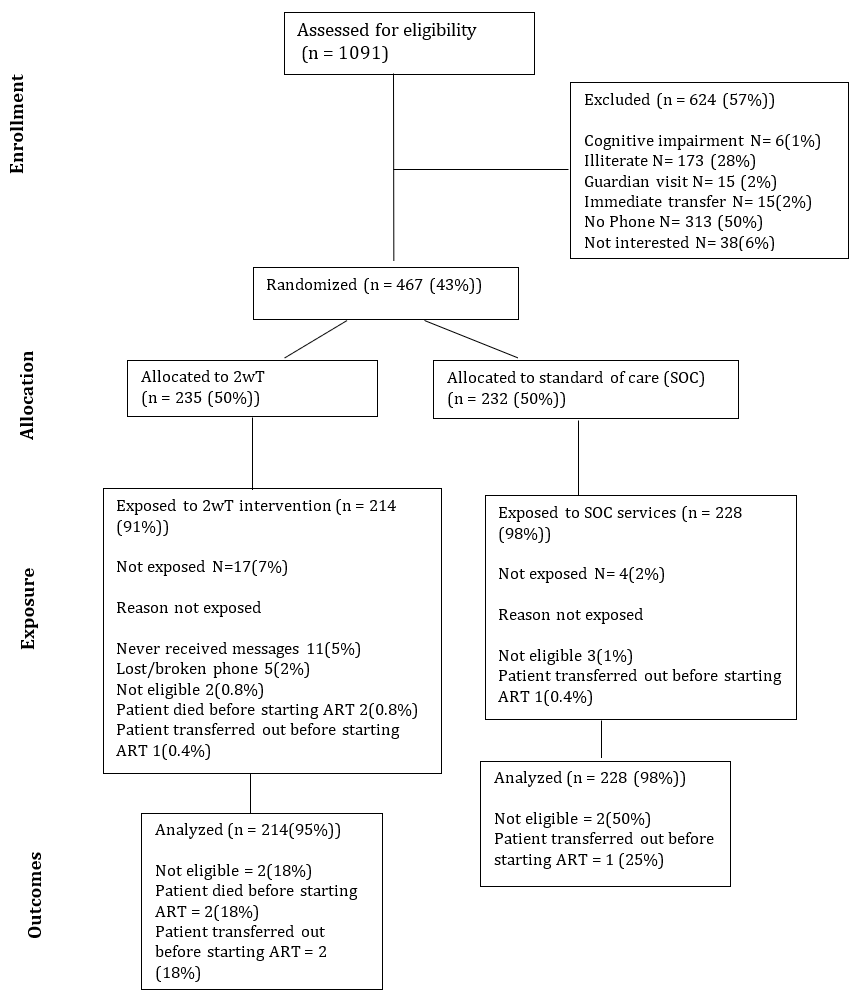

Supplement: S1 Fig — (DOCX) [file pgph.0004598.s003.docx]
