# Supplementary material for: Evaluating the effect of interactive two-way texting on 6-month antiretroviral therapy outcomes: Findings from a randomized controlled trial in Lilongwe, Malawi
Source: PLOS Glob Public Health. 2025 Sep 10;5(9):e0004598. doi: 10.1371/journal.pgph.0004598 (PMC12422422; doi:10.1371/journal.pgph.0004598)
Supplement: S2 Fig — (DOCX) [file pgph.0004598.s004.docx]

## ***S2 Fig: Kaplan-Meier Curve of Retention on ART among 2wT and SoC Clients Over Time***

The Kaplan-Meier curves revealed no difference in retention on ART over time between the 2wT and the SoC arm (p=0.4) (S2 Fig). Six months post-ART initiation, the probability of being retained on ART in the 2wT group was 97% (95% CI: 95% - 99%) compared to 96% (95%CI: 93% - 98%) in the SoC group.

**
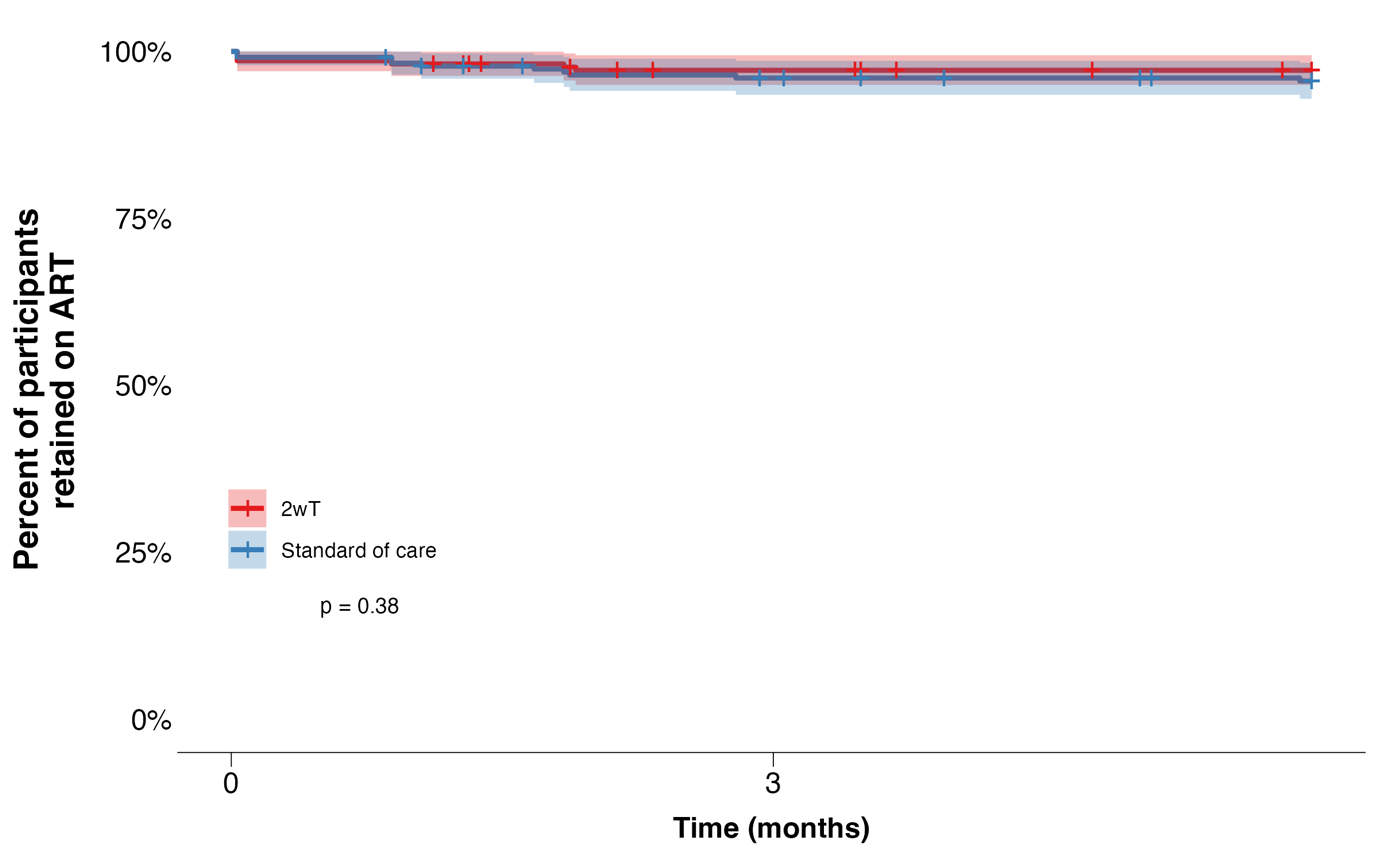
**
