# Supplementary material for: Evaluating the effect of interactive two-way texting on 6-month antiretroviral therapy outcomes: Findings from a randomized controlled trial in Lilongwe, Malawi
Source: PLOS Glob Public Health. 2025 Sep 10;5(9):e0004598. doi: 10.1371/journal.pgph.0004598 (PMC12422422; doi:10.1371/journal.pgph.0004598)
Supplement: S2 Table — (DOCX) [file pgph.0004598.s005.docx]

## ***S2 Table : ART outcome of Participants Without Viral Load Measurement at 6 Months***

Of the 442 participants, 123 (28%) did not have viral load measured at six months (S4 Table 2). Among them, 85 (68%) were alive on ART at 6 months, while the remaining 39 had disengaged from care before six months: 15 (12%) were LTFU, 1 (1%) stopped treatment, 17 (14%) transferred out, and 6 (5%) died. There was no significant difference between participants with and without VL in terms of age, age or WHO stage at initiation (all p-values > 0.1). The logistic model fit was poor (Pseudo R² = 0.017). Among the remaining 319 participants, the majority achieved VLS in both groups.

| ART outcome of Participants Without Viral Load Measurement at 6 Months (N = 123) | |
| --- | --- |
| **ART outcomes** | **N (%)** |
| Alive on ART at 6 months | 84 (68%) |
| Lost to follow-up | 15 (12%) |
| Stopped treatment | 1 (1%) |
| Transferred out | 17 (14%) |
| Died | 6 (5%) |
