# Supplementary material for: Evaluating the effect of interactive two-way texting on 6-month antiretroviral therapy outcomes: Findings from a randomized controlled trial in Lilongwe, Malawi
Source: PLOS Glob Public Health. 2025 Sep 10;5(9):e0004598. doi: 10.1371/journal.pgph.0004598 (PMC12422422; doi:10.1371/journal.pgph.0004598)
Supplement: S3 Table — (DOCX)s [file pgph.0004598.s006.docx]

## ***S3 Table: 2wT Reminder Messages by Appointment Timing***

All 214 participants (100%) received at least one three-day appointment reminder, 210 (98%) received at least one one-day reminder, while 162 (76%), 89 (42%), and 28 (13%) participants received reminders at two, five, and eleven days after a missed visit, respectively.

| **Reminder Timing** | **Number of Messages Sent by 2wT platform (N=1645) N (%)** |
| --- | --- |
| 3 days before appointment | 615 (37%) |
| 1 day before appointment | 597 (36%) |
| 2 days after missed appointment | 279 (17%) |
| 5 days after missed appointment | 115 (7%) |
| 11 days after missed appointment | 34 (2%) |
